# *Chlamydia trachomatis* Intercepts Golgi-Derived Sphingolipids through a Rab14-Mediated Transport Required for Bacterial Development and Replication

**DOI:** 10.1371/journal.pone.0014084

**Published:** 2010-11-22

**Authors:** Anahí Capmany, María Teresa Damiani

**Affiliations:** Cell Biology Laboratory, Biochemistry Department, IHEM-CONICET, Faculty of Medicine, University of Cuyo, Mendoza, Argentina; Duke University, United States of America

## Abstract

*Chlamydia trachomatis* are obligate intracellular bacteria that survive and replicate in a bacterial-modified phagosome called inclusion. As other intracellular parasites, these bacteria subvert the phagocytic pathway to avoid degradation in phagolysosomes and exploit trafficking pathways to acquire both energy and nutrients essential for their survival. Rabs are host proteins that control intracellular vesicular trafficking. Rab14, a Golgi-related Rab, controls Golgi to endosomes transport. Since *Chlamydia* establish a close relationship with the Golgi apparatus, the recruitment and participation of Rab14 on inclusion development and bacteria growth were analyzed. Time course analysis revealed that Rab14 associated with inclusions by 10 h post infection and was maintained throughout the entire developmental cycle. The recruitment was bacterial protein synthesis-dependent but independent of microtubules and Golgi integrity. Overexpression of Rab14 dominant negative mutants delayed inclusion enlargement, and impaired bacteria replication as determined by IFU. Silencing of Rab14 by siRNA also decreased bacteria multiplication and infectivity. By electron microscopy, aberrant bacteria were observed in cells overexpressing the cytosolic negative Rab14 mutant. Our results showed that Rab14 facilitates the delivery of sphingolipids required for bacterial development and replication from the Golgi to chlamydial inclusions. Novel anti-chlamydial therapies could be developed based on the knowledge of how bacteria subvert host vesicular transport events through Rabs manipulation.

## Introduction

*Chlamydia trachomatis* are Gram-negative obligate intracellular bacteria that constitute the leading cause of bacterial sexually transmitted diseases and preventable blindness worldwide [1], [2]. Chronic infections can result in several sequelae such us pelvic inflammatory disease, ectopic pregnancy and female infertility. *C. trachomatis* have a tropism for genital mucosal epithelium where they promote their uptake into nonphagocytic cells. Chlamydial infection and propagation rely upon a unique biphasic lifecycle which starts when infectious, environmentally resistant, elementary bodies (EBs) enter the cells within a membrane-bound vacuole termed the inclusion. Once inside this modified phagosome, EBs differentiate into metabolically active but non infectious reticulate bodies (RBs) that multiply by binary fission. After numerous rounds of replication, RBs undergo a transformation back into infectious EBs for spreading to adjacent cells [3], [4]. Indispensable to chlamydial growth and development is the establishment of an intracellular niche that avoids immune response but allows the acquisition of essential host nutrients [5]. To achieve this residency, these pathogens have evolved finely adapted mechanisms to circumvent the endocytic/lysosomal degradative route and for exploiting numerous intracellular trafficking pathways to acquire amino acids, nucleotides and lipids from the host cell [6]–[8]. It has been shown that chlamydial inclusions intersect TGN-derived vesicles [9]–[12], multivesicular bodies [13], [14] and lipid droplets [15], [16] for sphingomyelin, cholesterol and neutral lipids acquisition. At present, the strategies developed by *Chlamydia trachomatis* to re-route intracellular trafficking are being actively studied.

Rab GTPases, the masters in control of intracellular vesicular transport and organelle identity, have been implicated in chlamydial inclusion development [17]. Rab proteins are small GTPases that cycle between an active prenylated GTP-bound form and an inactive cytosolic GDP-bound form. The Rab family includes almost 70 members and each one is implicated in the control of a defined transport step [18], [19]. It has been reported that Rab1, Rab4 and Rab11 are recruited to the inclusion membrane from all chlamydial species, whereas Rab6 and Rab10 are associated with inclusions in a species-specific manner [20]–[22]. It has been recently shown that silencing of Rab6 and Rab11 by siRNA impaired lipid acquisition and replication of *C. trachomatis* [21]. Conveniently, classical Rabs belonging to the endocytic/phagocytic pathway like Rab5 and Rab7 are quickly excluded from the membrane inclusion [6], [8]. This selective recruitment of Rab proteins may serve not only for camouflaging the inclusion from lysosomes, but also for advantageously targeting host organelles and vesicles full of nutrients to chlamydial inclusions.

Rab14, a GTPase involved in the delivery of TGN-derived vesicles to endosomes and the plasma membrane, has recently been described [23]–[25]. This protein has been pointed out to contribute to *Mycobacterium tuberculosis*-containing phagosomes maturation arrest [26]. A Rab14 homologous in *Dyctiostelium discoideum*, RabD, participates in homotypic phagosome fusion [27]. The present study shows and characterizes the recruitment of Rab14 to *Chlamydia trachomatis*-containing inclusions. Furthermore, our data show the beneficial impact of Rab14 on chlamydial inclusion development, sphingolipid transport and bacteria replication.

## Results

### Rab14 associates to chlamydial inclusions

**Figure 1:**
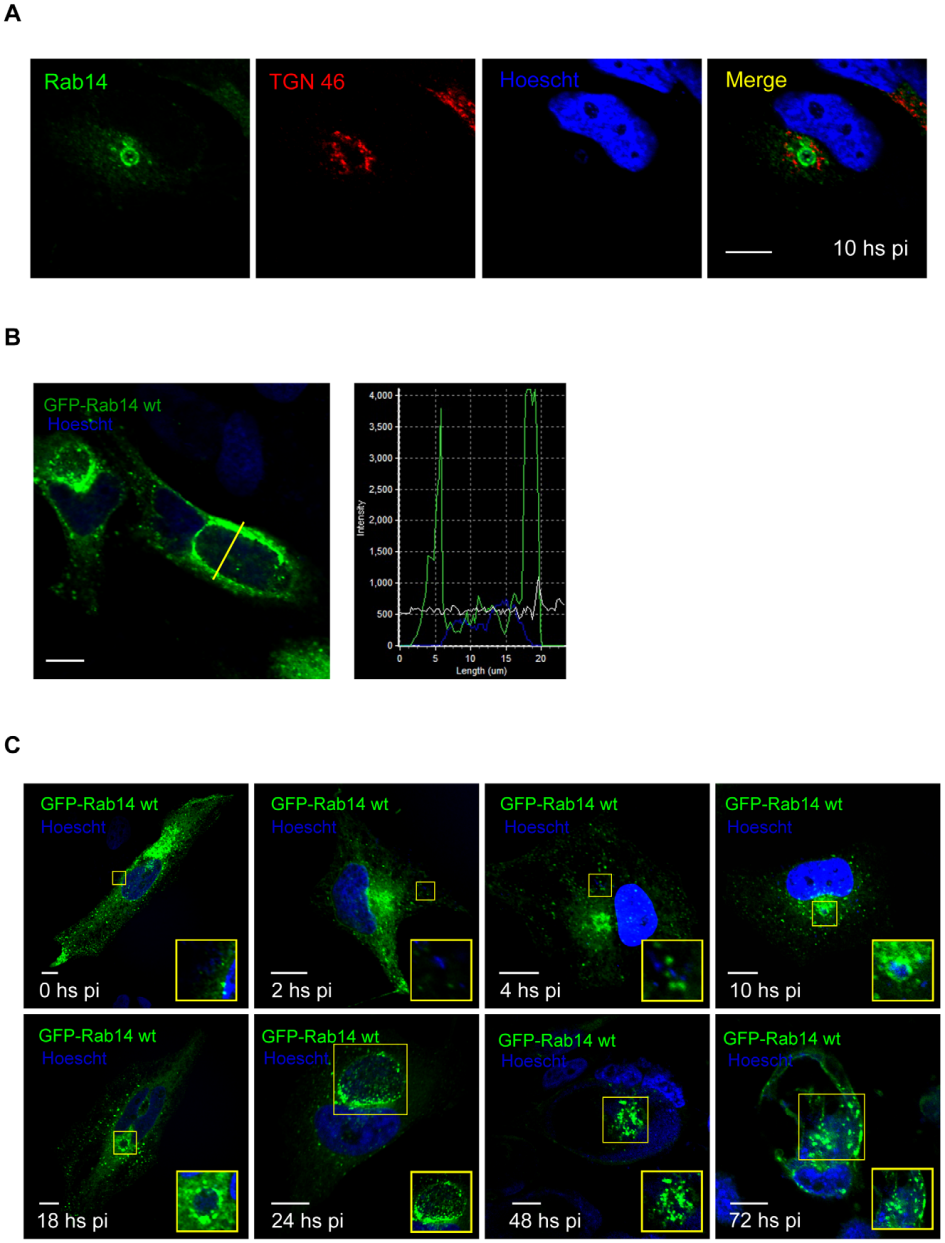
Rab14 is associated with chlamydial inclusions. **A**) HeLa cells were infected with *C. trachomatis* serovar L2 (MOI 5) for 10 h. Cells were fixed, permeabilized and stained with rabbit polyclonal anti-Rab14 (1∶100) followed by FITC-labeled goat anti-rabbit IgG (1∶200) (green). Bacterial DNA was evidenced by Hoescht staining (blue). Golgi apparatus was stained with mouse monoclonal anti-TGN 46 antibodies (1∶200) followed by Cy5-conjugated anti-mouse IgG (1∶700) (red) Endogenous Rab14 was clearly recruited to the inclusion. **B**) HeLa cells overexpressing GFP-Rab14wt were infected with *C. trachomatis* serovar L2 (MOI 5) for 24 h and analyzed by confocal microscopy. The intensity profile showed GFP-Rab14wt (green) on the inclusion membrane surrounding the Hoescht-labeled bacteria (blue). **C**) HeLa cells overexpressing GFP-Rab14wt (green) were infected with *C. trachomatis* serovar L2 and fixed at indicated p.i. times. Bacterial and eukaryotic DNA was labeled with Hoescht (blue). Insets show a magnification of the marked area. Bar 10 µm.

*Chlamydia trachomatis* establish a close relationship with the Golgi apparatus of the infected host cell [21], [28]. To date, it has been reported that several Rabs involved in Golgi-related transport events are specifically recruited to chlamydial inclusions. Rab1, which controls endoplasmic reticulum to Golgi trafficking, and Rab4 and Rab11, which regulate transport from the endocytic recycling compartment (ERC) and from endosomes to the trans-Golgi (TGN), associate with inclusions from all the chlamydial species tested [20]. On the other hand, Rab6, that rules Golgi to endoplasmic reticulum retrograde trafficking, is present only in *C. trachomatis*-containing inclusions; whereas Rab10, another Golgi-associated GTPase, is recruited exclusively to *C. pneumoniae* inclusions [17], [29]. To determine whether Rab14, a Rab that regulates TGN to endosomes and plasma membrane transport [23], [25], localized to chlamydial inclusions, we examined intracellular distribution of endogenous Rab14 in infected cells. HeLa cells were infected with *C. trachomatis* serovar L2, fixed at 10 h p.i. and processed to detect endogenous Rab14 with a specific antibody followed by a FITC-labeled secondary IgG (green). Bacteria were evinced by Hoescht DNA staining (blue). Additionally, cells were labeled with mouse monoclonal anti-TGN 46, a marker for the Golgi complex, and the appropriated anti-mouse Cy5-conjugated secondary antibodies to visualize Golgi localization (red). Cells were viewed by laser-scanning confocal microscopy (LSCM). The immunofluorescence confocal images clearly showed endogenous Rab14 surrounding chlamydial inclusions (Figure 1A). Rab14 decorated the periphery of the inclusion in a defined rim-like staining pattern. Endogenous Rab14 delimiting the chlamydial inclusions were observed in the totality of the infected cells once the inclusion had arrived to the perinuclear region. This finding is of great interest since previous published reports have described the association of several Rabs (Rab1, 4, 6, 10 and 11) to chlamydial inclusions by using GFP-tagged overexpressed Rab constructs [20]–[22].

Since Rab proteins fused to GFP have been shown to colocalize with their respective endogenous proteins, HeLa cells overexpressing GFP-Rab14 were used for the subsequent experiments. The association of Rab14 to the chlamydial inclusion was further confirmed by the analysis of the distribution of GFP-Rab14wt (green) and Hoescht-labeled bacteria (blue) along a line traversing the chlamydial inclusion. Hoescht labels eukaryotic and bacterial DNA. The intensity profile showed Hoescht-labeled bacteria between two pikes of GFP-Rab14wt (Figure 1B). The presence of Rab14 associated with chlamydial inclusions was further confirmed by the analysis of z-sections through the center of the inclusion. The confocal planes revealed a fine punctuate pattern of GFP-Rab14wt surrounding the inclusion with Hoescht-labeled *Chlamydia* inside (Figure S1A). Several y sections from a 3D-reconstruction of the z-optical planes are shown in Figure S1B.

The temporal expression pattern of *Chlamydia trachomatis* genes varies along their developmental cycle [3]–[5]. As a consequence, a differential timing has been demonstrated in the association of Rab GTPases with the chlamydial inclusion. To analyze the recruitment of Rab14 to the inclusions along the developmental cycle, HeLa cells overexpressing GFP-Rab14wt were infected with *C. trachomatis* and fixed at different post-infection (p.i.) times. At the initial stages of infection (2 h and 4 h p.i.) no GFP-Rab14wt surrounding the nascent inclusions was observed. The recruitment of GFP-Rab14wt was evident at 10 h p.i. when the vacuoles had arrived at their peri-nuclear localization. The amount of GFP-Rab14wt associated to chlamydial inclusions rose during the mid-stage developmental cycle (18 h to 24 h p.i.) showing a rim-shape staining pattern delimiting the inclusions. GFP-Rab14wt recruitment was maintained throughout the entire developmental cycle. At later stages of infection (48 h to 72 h p.i.) some GFP-Rab14wt remained associated with the inclusion membrane but surprisingly, most GFP-Rab14wt label was observed inside chlamydial inclusions (Figure 1C). At the final stages of infection, the inclusion occupied almost the entire cell and the majority of intracellular GFP-Rab14wt was found associated with the chlamydial inclusion whereas the other intracellular stores of this Rab were depleted.

Rab GTPases cycle between an active membrane-associated GTP-bound form and an inactive cytosolic GDP-bound form. Usually GTP-charged Rabs are associated with membranes through isoprenoid molecules added at their C-terminus. This hydrophobic tail is hidden when the Rab is bound to GDP, turning the Rab cytosolic and associated to a chaperon protein named GDP Dissociating Inhibitor (GDI) [18], [19]. However, this general model for Rabs behavior is being challenged by increasing evidence supporting that GDP/GTP cycles can also occur on membranes without recycling to the cytosol. An example is the GDP-bound form of Rab11 which, by the formation of a symmetrical dimmer, remains associated with membranes and could interact with certain Rab11 partners [30]. In uninfected HeLa cells, GFP-Rab14wt distributed to its appropriate subcellular localization showing a punctuate pattern dispersed throughout the cytosol and mostly concentrated at the Golgi apparatus. Its inactive GDP-bound form, GFP-Rab14S25N, is mostly anchored to membranes and trapped at the Golgi apparatus whereas another negative non-prenylated mutant, GFP-Rab14ΔGCGC, is exclusively diffuse and cytosolic (Figure S2).

**Figure 2:**
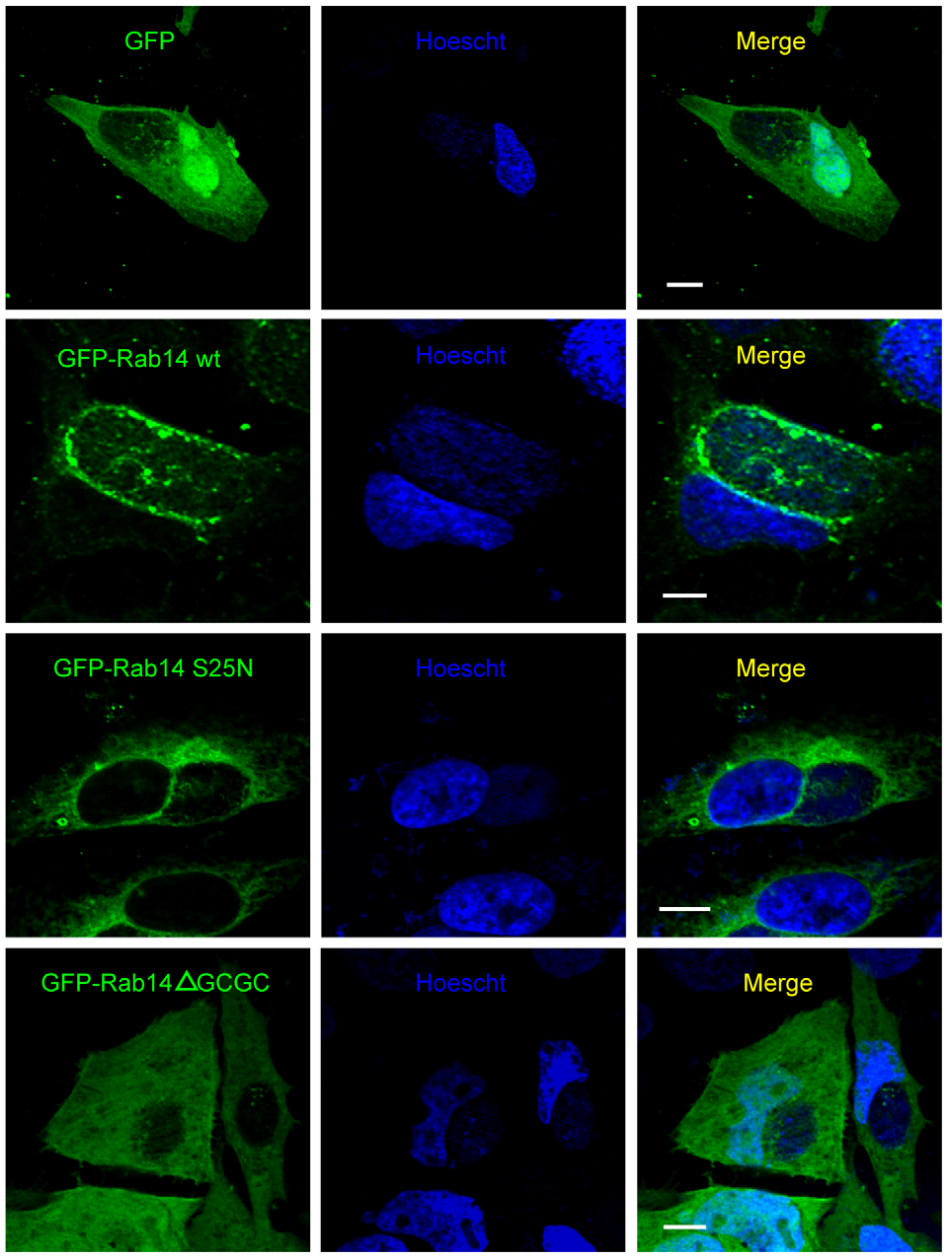
Rab14ΔGCGC is not recruited to chlamydial inclusions. HeLa cells were transfected with pEGFP, pEGFP-Rab14wt, pEGFP-Rab14S25N and pEGFP-Rab14ΔGCGC and 24 h later were infected with *C. trachomatis* serovar L2 (MOI 5). Cells were fixed at 24 h p.i. and bacterial DNA was stained with Hoescht (blue). GFP-Rab14wt (green) was localized at the chlamydial inclusion membrane. Confocal images are representative of four independent experiments. Bar 10 µm.

To assess the distribution of GFP-Rab14wt and its mutants in infected cells, transfected HeLa cells were infected with *C. trachomatis* and fixed 24 h later. Confocal images showed that GFP-Rab14wt decorated the chlamydial inclusion whereas its negative mutant GFP-Rab14ΔGCGC was not found associated with the inclusion and remained cytosolic. GFP-Rab14S25N, the negative GDP-anchored mutant, was retained at the Golgi apparatus in the vicinity of the vacuole (Figure 2). Rab14 recruitment was independent of the MOI used, since we have observed Rab14 associated to inclusions in cells infected at an MOI of less than 1 or more than 20.

**Figure 3:**
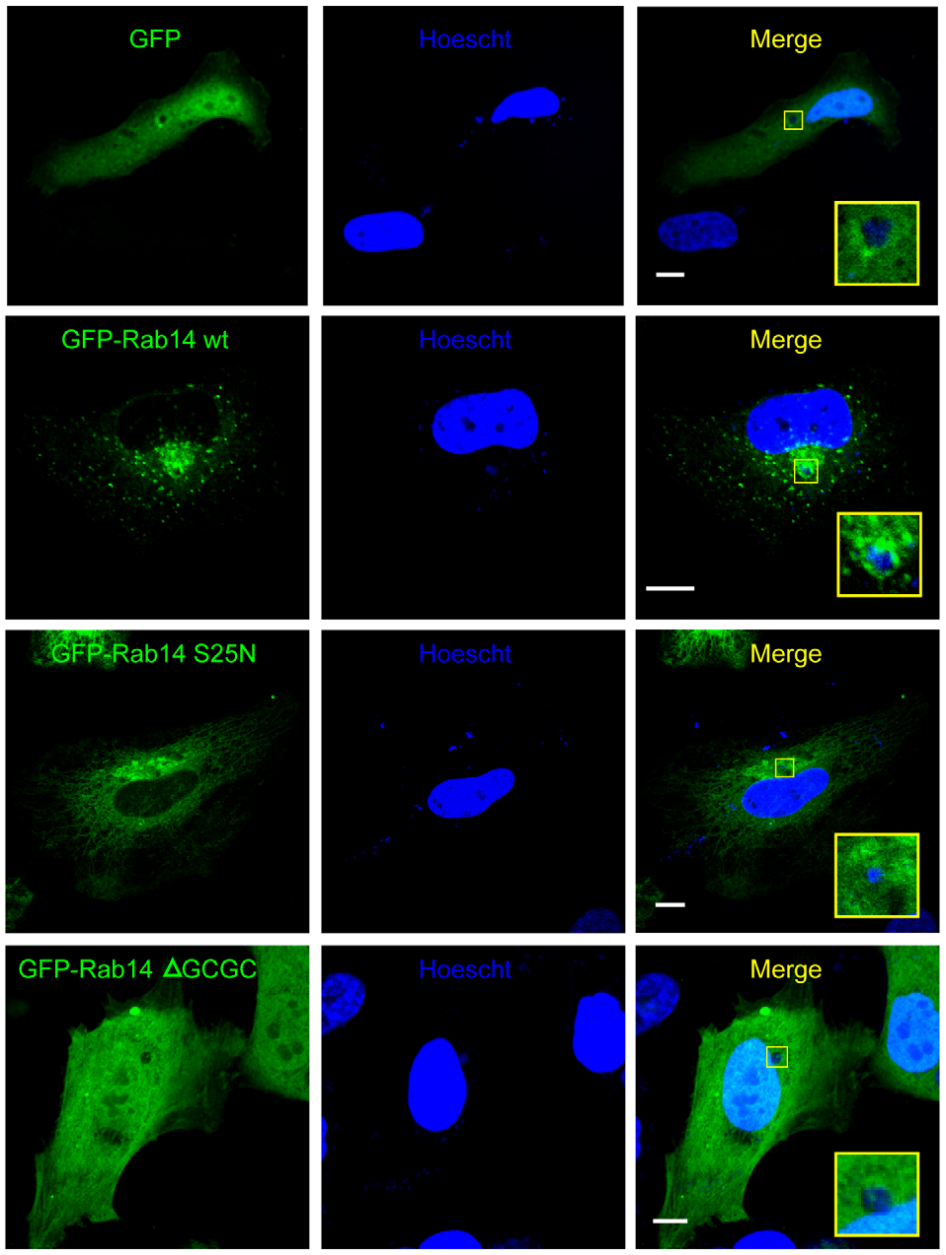
Rab14 recruitment to early chlamydial inclusions. HeLa cells overexpressing GFP, GFP-Rab14wt, GFP-Rab14S25N or GFP-Rab14ΔGCGC (green) were infected with *C. trachomatis* serovar L2 (MOI 5) and fixed at 10 h p.i. Bacterial and eukaryotic DNA was stained with Hoescht (blue). Insets show a magnification of the selected area of the cell. GFP-Rab14wt was recruited to the chlamydial inclusion membrane, whereas the negative GDP-bound mutant Rab14S25N and the negative cytosolic mutant Rab14ΔGCGC were not associated to the inclusion. Confocal images are representative of three independent experiments. Bar 10 µm.

**Figure 4:**
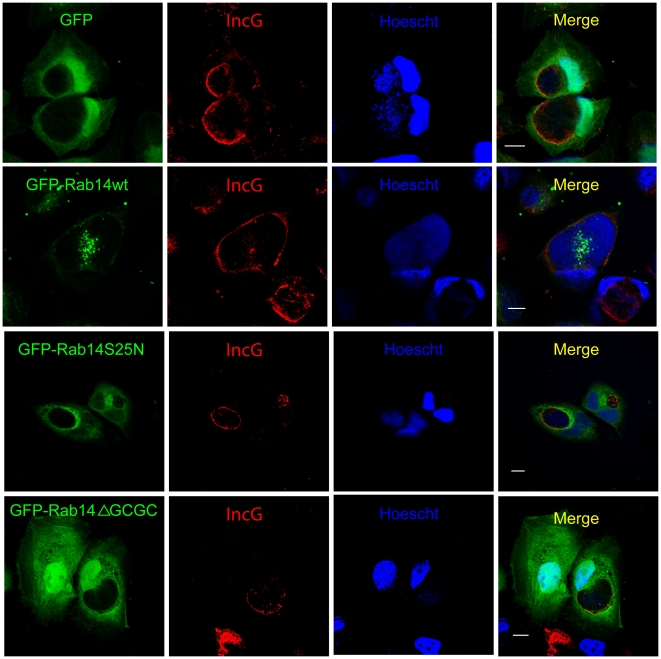
Rab14 recruitment to late chlamydial inclusions. HeLa cells were transfected with pEGFP, pEGFP-Rab14wt, pEGFP-Rab14S25N and pEGFP-Rab14ΔGCGC (green) and 24 h later were infected with *C. trachomatis* serovar L2 (MOI 5). Cells were fixed at 48 h p.i. and bacterial DNA was stained with Hoescht (blue). Inclusion membrane was labeled with polyclonal rabbit anti-IncG antibodies (1∶20) followed by goat anti-rabbit Texas Red-conjugated antibodies (1∶200) (red). Note GFP-Rab14wt-labeled vesicular structures inside inclusions (green). Images are representative of five independent experiments. Bar 10 µm.

To further analyze the recruitment of Rab14 to chlamydial inclusions, Hela cells overexpressing GFP-Rab14wt and its mutants were infected with *C. trachomatis* and fixed at 10 h p.i. Confocal images showed a clear association of GFP-Rab14wt (green) with perinuclear chlamydial inclusions at early stages of development. A well-defined ring of GFP-Rab14wt delimiting the bacterial inclusion was observed, whereas no association of the negative mutants neither the GDP-bound form (GFP-Rab14S25N) nor the non-prenylated cytosolic protein (GFP-Rab14ΔGCGC) was appreciated (Figure 3). In order to gain deeper insight into the recruitment of Rab14 to chlamydial inclusions, another group of infections were carried out under similar conditions but cells were fixed at 48 h p.i. Interestingly we found intrainclusion structures labeled with Rab14, mainly in cells overexpressing GFP-Rab14wt. In contrast, cells overexpressing the negative non-prenylated mutant GFP-Rab14ΔGCGC displayed inclusions without Rab14-labeling inside, as well as, with less blue fluorescence associated to Hoescht-labeled bacterial DNA. To delimit better the chlamydial inclusion edges, cells were stained with rabbit polyclonal anti-IncG antibodies followed by anti-rabbit Texas Red-labeled secondary IgG. IncG is a bacterial protein expressed early in the bacterial developmental cycle that serves as a trustworthy marker of inclusion membrane (Figure 4). These GFP-Rab14wt positive structures observed in the inclusion lumen by confocal microscopy resembled intrainclusion vesicles. The three-dimensional reconstruction of GFP-Rab14wt overexpressing cells (green) infected with *C. trachomatis* (blue) for 48 h and labeled with anti-IncG antibodies (red) showed vesicular structures decorated by GFP-Rab14wt within the limits of the inclusions close to the bacteria (Video S1). Images obtained by transmission electron microscopy (TEM) of infected GFP-Rab14wt overexpressing cells fixed at 48 h p.i. showed membranous structures inside chlamydial inclusions (Figure S3). Ongoing experiments are being conducted to characterize these vesicular structures found into the inclusion lumen of GFP-Rab14wt overexpressing cells at late stages of chlamydial infection.

Taken together, these results showed that a functional Rab14 was recruited to the chlamydial inclusion. This recruitment could be mediated through an interaction with a host or bacterial Rab14 binding molecule located at the chlamydial inclusion membrane.

### Rab14 recruitment is bacterial protein synthesis-dependent but independent of IncA exportation

**Figure 5:**
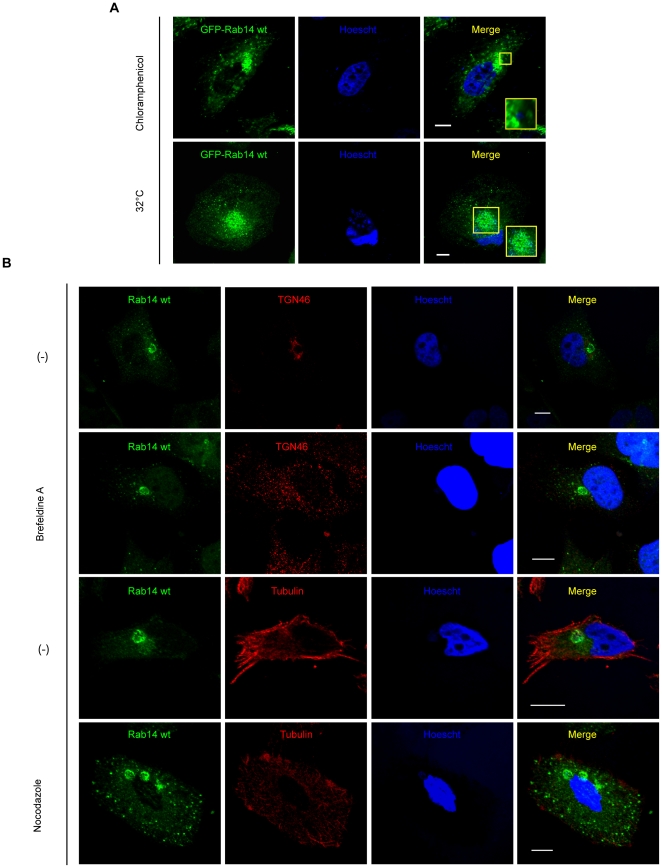
Characterization of Rab14 recruitment. HeLa cells overexpressing GFP-Rab14wt (green) were infected with *C. trachomatis* serovar L2 (MOI 10) and incubated with: **A**) 200 µg/ml Chloramphenicol (20 h) (upper panels) or at 32°C (24 h) (lower panels); and **B**) HeLa cells infected with *C. trachomatis* L2 (MOI 10) were incubated with 1 µg/ml Brefeldin A (6 h) (upper panels) or 20 µM Nocodazole (6 h) (lower panels), prior fixation at 10 h p.i. Bacteria were labeled with Hoescht (blue). Endogenous Rab14 was detected using rabbit polyclonal anti-Rab14 (1∶100) followed by goat anti-rabbit FITC-labeled antibodies (1∶200) (green). Mouse monoclonal anti-TGN 46 or mouse monoclonal anti-β-tubulin followed by donkey anti-mouse Cy5-labeled antibodies (1∶700) (red) were used to detect Golgi apparatus or microtubules, respectively. The data are representative of three independent experiments. Bar 10 µm.

It has been shown that once inside cells, early chlamydial protein synthesis is needed for inclusion remodeling to avoid fusion with lysosomes and for inclusion trafficking to the pericentriolar region of the host cell [31]. To determine whether Rab14 recruitment to the inclusion also requires bacterial protein synthesis, HeLa cells overexpressing GFP-Rab14wt were infected with *Chlamydia trachomatis.* After the first two hours corresponding to the uptake period, 200 µg/ml of chloramphenicol was added for the following 24 h. Then, cells were fixed and analyzed by confocal microscopy. As shown in Figure 5A upper panels, under chloramphenicol treatment, *Chlamydia*-containing vacuoles remained dispersed throughout the host cytosol without association with GFP-Rab14wt. These results demonstrated that chlamydial protein synthesis was required for Rab14 recruitment to chlamydial inclusion. Therefore, Rab14 targeting to the inclusion is an active mechanism exerted by the bacteria for the remodeling of their intracellular niche.

An increasing number of studies explore the association of host Rabs with chlamydial inclusions. Nevertheless, the bacterial proteins that participate in such interaction remain mostly unknown. A family of integral inclusion membrane proteins named Incs is pointed out as the bacterial molecules responsible for the binding of host Rabs to the inclusion. The CT229, a *C. trachomatis* Inc, specifically binds Rab4 [22]; whereas Cpn0585, a *C. pneumoniae* inclusion membrane protein, interacts with Rab1, Rab10, and Rab11 [32]. Another Inc protein, IncA, displays a SNARE-like motif [33] and is involved in homotypic fusion of inclusions [34], [35]. Recently, it has been shown in an *in vitro* liposome fusion assay that IncA interferes with the SNARE-mediated membrane fusion [36]. In addition, *Chlamydia trachomatis*-containing inclusions do not fuse with each other when infected cells were cultivated at 32°C. This observation correlates with restricted export of IncA [34], [35]. To assess whether Rab14 was recruited to chlamydial inclusion through IncA, infected HeLa cells overexpressing GFP-Rab14wt were maintained at 32°C during 24 h. Multiple small non-fused inclusions (some of them corresponding to individual bacterium-containing vacuoles) were formed, but still strongly decorated with GFP-Rab14wt (Figure 5A lower panels). These results showed that the recruitment of Rab14 to the inclusion was independent of the exposition of IncA or another temperature-sensitive exported Inc to the inclusion membrane. In addition, Rab14 recruitment was also independent of the formation of a large unique single inclusion typical of *C. trachomatis*-infected cells cultivated at 37°C.

### Golgi disruption does not impede Rab14 recruitment

Rab14 has been implicated in the delivery of TGN-derived vesicles to early endosomes and the plasma membrane [23]–[25]. In order to determine whether an intact Golgi apparatus was required for the localization of Rab14 to the chlamydial inclusion, cells were treated with Brefeldine A (BFA), a fungal metabolite that causes the collapse of the Golgi apparatus into the ER. HeLa cells infected with *C.trachomatis* for 10 h were incubated with 1 µg/ml BFA the last 6 h before fixation. Endogenous Rab14 was detected using specific anti-Rab14 antibodies followed by FITC-labeled secondary IgG (green). Golgi apparatus was evinced by anti-TGN 46 antibodies followed by the appropriated Cy5-conjugated secondary antibodies (red). Bacterial and eukaryotic DNA was labeled with Hoescht (blue). As shown in Figure 5B upper panels, endogenous Rab14 was recruited to chlamydial inclusions, as demonstrated by the well-defined rim-like green fluorescence surrounding each of the inclusions even after Golgi disorganization by BFA treatment. Similar results were obtained using GFP-Rab14wt overexpressing cells infected with *C. trachomatis* and treated with 1 µg/ml BFA during the last six hours until fixation at 24 h p.i. Golgi apparatus disruption was confirmed by staining with anti-TGN 46 antibodies. Confocal images showed a clear association of GFP-Rab14wt to chlamydial inclusions even when the Golgi apparatus was disrupted (Figure S4 upper panels). Collectively, these results demonstrate that the recruitment to chlamydial inclusions of both, endogenous Rab14 and overexpressed GFP-Rab14wt is independent of Golgi apparatus integrity.

*Chlamydia trachomatis* are actively trafficked to the pericentriolar region of the cell depending on host cytoskeleton [37]. Likewise, an intact microtubule network is required for Golgi apparatus integrity and its perinuclear localization [38]–[40]. It has been shown that ERC-associated Rab4 and Rab11, as well as Golgi-localized Rab1, Rab6 and Rab10, remained associated with chlamydial inclusions after microtubule disruption by nocodazole treatment [20]–[22]. To assess if the localization of Rab14 surrounding the inclusion requires intact microtubules, HeLa cells infected with *C. trachomatis* were incubated with 20 µm Nocodazole for 6 h before fixation at 10 h p.i. The absence of an intact microtubule network in Nocodazole-treated cells was determined by indirect immunofluorescence using a mouse monoclonal anti-β-tubulin antibody followed by donkey anti-mouse Cy5-conjugated secondary antibodies. Endogenous Rab14 distribution was assessed by using rabbit polyclonal anti-Rab14 antibodies and the appropriated FITC-labeled secondary antibodies. Bacteria were evinced by Hoescht DNA staining. Confocal images showed that endogenous Rab14 association with chlamydial inclusions was unaffected by treatment with the microtubule-destabilizing drug Nocodazole as indicated by the distinct rim-like staining pattern surrounding the chlamydial inclusions (Figure 5B lower panels). Coincidently, most GFP-Rab14wt remained associated with the inclusion despite microtubules depolymerization in GFP-Rab14wt overexpressing cells infected with *Chlamydia trachomatis* and treated with 20 µm Nocodazole for 12 h prior fixation at 24 h p.i. (Figure S4 lower panels). Therefore, both approaches indicated that Rab14 was trafficked to the inclusion by a mechanism independent of microtubules dynamics. Similar results were obtained using Vinblastine, another microtubule-depolymerizing drug (data not shown). Since depolymerization of microtubules provokes the dispersion of Golgi stacks throughout the cytosol, these data also confirmed that Rab14 recruitment to the inclusion was independent of both, Golgi apparatus integrity and an intact cytoskeleton dynamics.

### Rab14 favors inclusion growth and bacteria replication

**Figure 6:**
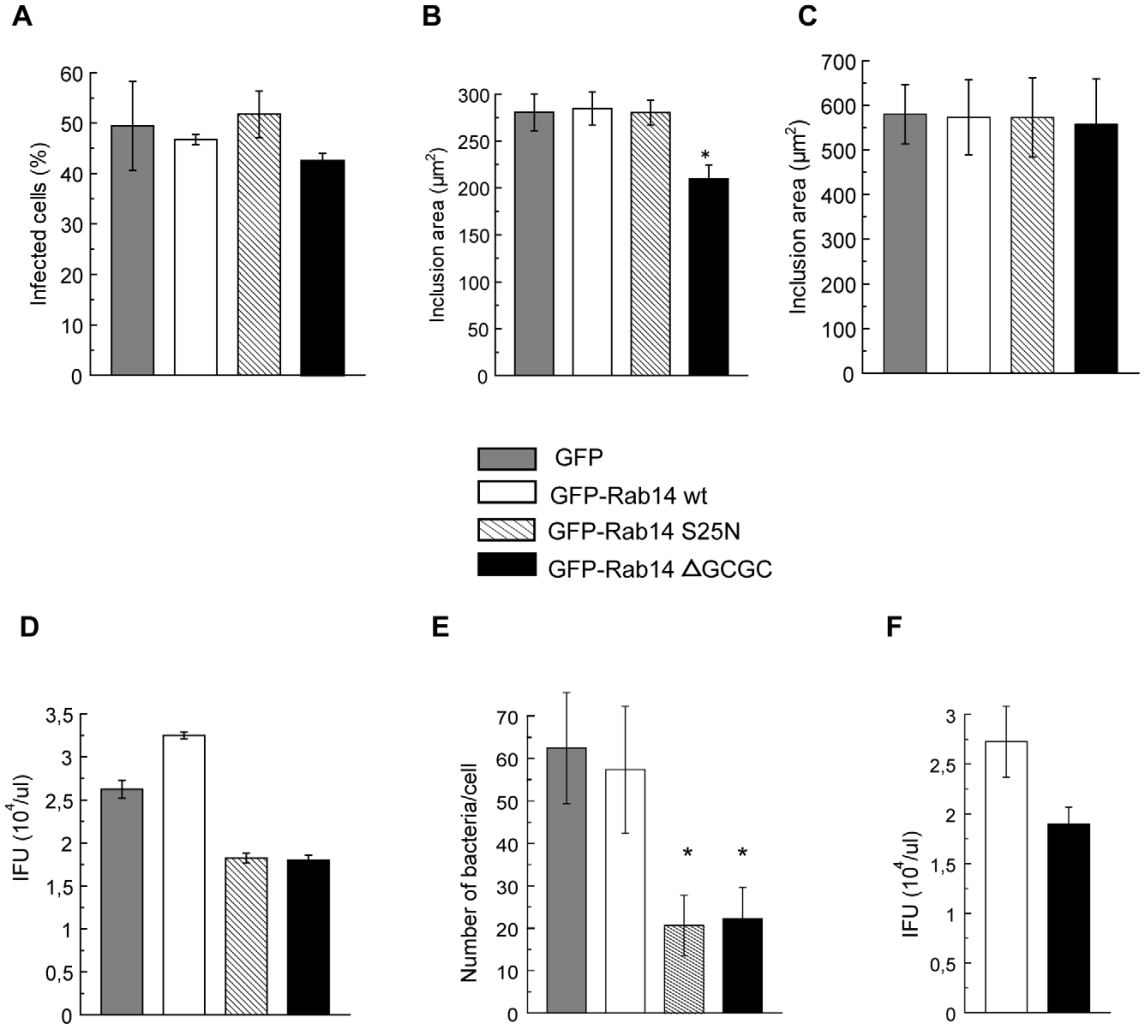
Rab14 favors inclusion growth and bacteria replication. HeLa cells overexpressing GFP, GFP-Rab14wt or the mutants GFP-Rab14S25N or GFP-Rab14ΔGCGC were infected with *C. trachomatis* L2 at a MOI of 10. **A**) Percentage of infected cells fixed at 10 h p.i. and quantified by confocal microscopy. Bacterial DNA was labeled with Hoescht. **B**) Chlamydial inclusion area from cells overexpressing GFP alone, GFP-Rab14 wt or both negative mutants GFP-Rab14S25N or GFP-Rab14ΔGCGC infected with *C. trachomatis* L2 at a MOI of 5 and fixed 24 h p.i. Inclusion area was measured by confocal microscopy. Results are expressed as the mean ± sem. **C**) Area of inclusions assessed by confocal microscopy. Cells overexpressing GFP alone, GFP-Rab14 wt or both negative mutants GFP-Rab14S25N or GFP-Rab14ΔGCGC were infected with *C. trachomatis* L2 at a MOI of 5 and fixed 48 h p.i. Results are expressed as the mean ± sem. In Panels A to C, at least 50 cells of each condition coming from three independent experiments were analyzed. Results were statistically analyzed by one-way ANOVA and Tukey post-test (*p<0.05). **D**) Infected HeLa cells overexpressing GFP, GFP-Rab14wt, GFP-Rab14S25N or GFP-Rab14ΔGCGC were lysed at 48 h p.i. (MOI of 5). The infectious particles (EBs) released were titrated on fresh HeLa cells by counting the Inclusion Forming Units (IFU) 24 h later as described in Material and Methods. The graph represents media ± sem of three independent experiments performed in duplicates. **E**) HeLa cells overexpressing GFP, GFP-Rab14wt, GFP-Rab14S25N or GFP-Rab14ΔGCGC infected with *C. trachomatis* for 24 h were fixed and processed for Transmission Electron Microscopy (TEM). Bacteria inside inclusions were counted in 30 cells of each condition from five independent experiments. Data were statistically analyzed by one-way ANOVA and Tukey post-test (*p<0.05). **F**) HeLa cells transfected with negative control siRNA (open bar) or specific siRNA against human RAB14 (black bar) were infected with *C. trachomatis* (MOI of 5) at 72 h post transfection. Then, cells were lysed at 48 h p.i. The infectious particles (EBs) released were titrated on fresh HeLa cells by counting the Inclusion Forming Units (IFU) 24 h later as described in Material and Methods. The graph represents media and range of two independent experiments performed in duplicates.

GFP-Rab14 was not associated to nascent inclusions, suggesting that this GTPase was not involved in the entry process. To analyze the role of Rab14 on bacteria internalization, transfected HeLa cells were infected with *C. trachomatis* and fixed at 10 h p.i. The percentage of infected cells was unaffected by the overexpression of GFP alone, GFP-Rab14wt or both negative mutants GFP-Rab14S25N or GFP-Rab14ΔGCGC (Figure 6A). Likewise, the number of initial inclusions formed was not modified (data not shown). In contrast, the enlargement of chlamydial inclusions along time was significantly delayed in the presence of the negative cytosolic GFP-Rab14ΔGCGC mutant. This effect was evident at the mid-stage inclusions assessed by confocal microscopy. Overexpression of the negative cytosolic mutant GFP-Rab14ΔGCGC reduced the chlamydial inclusion size without affecting the number of inclusions per cell or the bacteria internalization rate at 24 h p.i. (Figure 6B). These data were in agreement with the results obtained by measuring inclusion diameter by TEM (data not shown). However, at later stages of development, chlamydial inclusions from all cells (GFP, GFP-Rab14wt, and both negative mutants GFP-Rab14S25N and GFP-Rab14ΔGCGC overexpressing cells) reached a similar size (Figure 6C).

To address the effect of Rab14 on bacterial growth and replication, infected HeLa cells overexpressing GFP, GFP-Rab14wt, GFP-Rab14S25N or GFP-Rab14ΔGCGC were lysed at 48 h p.i. The infectious particles (EBs) released were titrated on fresh HeLa cells by counting the Inclusion Forming Units (IFU) 24 h later. The amount of infectious bacteria recovered from GFP-Rab14S25N or GFP-Rab14ΔGCGC overexpressing cells was diminished compared to GFP-Rab14wt and GFP overexpressing cells (Figure 6D). The IFU assays evinced the adverse impact of Rab14 negative mutants on bacteria multiplication. In addition, quantification of bacteria inside inclusions revealed a reduction in the mean number of bacteria per cell inside both Rab14 negative mutants overexpressing cells compared to control cells, assessed by transmission electron microscopy (TEM) at 24 h p.i. (Figure 6E).

To deeper analyze the effect of host Rab14 GTPase on *C. trachomatis* replication, the endogenous protein synthesis was silenced by RNA interference. Briefly, HeLa cells were transfected with chemically synthesized short interfering RNA (siRNA) targeted to Rab14 or negative control siRNA. At 72 h post transfection, cells were infected with *C. trachomatis.* Then, cells were lysed at 48 h p.i. and infectious particles were titrated on fresh HeLa cells by IFUs assays. Silencing of Rab14 substantially reduced bacterial progeny in comparison to control cells (Figure 6F). These results further confirmed the requirement of a functional Rab14 for bacterial development and replication. Knockdown of Rab14 (more than 70%) was confirmed by western blot using rabbit polyclonal anti-Rab14 antibodies and goat anti-rabbit HRP-conjugated IgG (Figure S5). The detrimental impact on bacterial multiplication observed after Rab14 silencing or after Rab14 negative mutants overexpression was similar, suggesting a Rab14-specific effect.

**Figure 7:**
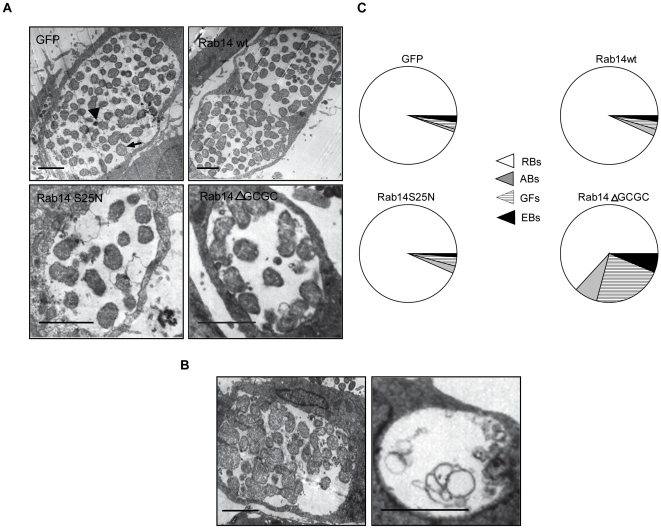
Ultrastructural analysis of chlamydial inclusions. **A**) HeLa cells overexpressing GFP, GFP-Rab14wt, GFP-Rab14S25N and GFP-Rab14ΔGCGC infected with *C. trachomatis* serovar L2 (MOI 5) were fixed at 24 h p.i. and processed for TEM. The arrow head shows an EB (smaller and electron dense) and the large arrow shows a RB (larger and less electron dense). Bar 2.25 µm. **B**) GFP-Rab14ΔGCGC overexpressing cells infected with *C. trachomatis* serovar L2 (MOI 5) for 24 h. Left panel shows an inclusion containing enlarged abnormal chlamydial developmental forms. Right panel shows an inclusion containing membrane structures and ghost bacterial forms. Bar 2.25 µm. **C**) Pie graphs show the relative proportion of reticulate bodies (RBs), elementary bodies (EBs) and large aberrant bacteria (ABs) and ghost bacterial forms (GFs) inside inclusions from GFP, GFP-Rab14wt, GFP-Rab14S25N and GFP-Rab14ΔGCGC overexpressing cells infected with *C. trachomatis* serovar L2 (MOI 5) for 24 h analyzed by TEM. At least 20 cells of each condition from three independent experiments were quantified.

The effect of Rab14 on *Chlamydia trachomatis* developmental cycle was assessed by the analysis of inclusions from HeLa cells overexpressing GFP, GFP-Rab14wt and its negative mutants at the ultrastructural level by transmission electron microscopy (TEM). The transfection efficiency of HeLa cells ranged between 85 to 95% with all plasmids used as shown in Figure S6. These allow us to use transiently transfected cells for TEM and IFU analysis. Since transfected cells could not be distinguish from un-transfected ones by TEM, the totality of the cells present on the grids were considered for ultrastructural analysis and bacterial quantification. The two chlamydial developmental forms (EBs and RBs) can be distinguished by TEM: the infectious EBs are smaller and electron dense, whereas RBs are less electron dense and larger. *Chlamydia-*infected cells developed a huge unique inclusion containing mainly RBs at 24 h p.i. and primarily EBs by 48 h p.i. Images from GFP-Rab14wt expressing cells revealed a normal chlamydial developmental cycle compared to GFP control cells. In contrast, TEM images showed altered bacterial developmental forms mainly in the negative cytosolic mutant overexpressing cells (Figure 7A). The presence of markedly enlarged, atypical chlamydial forms, distinct from both EBs and RBs was observed inside inclusions from cells overexpressing the negative cytosolic mutant of Rab14, GFP-Rab14ΔGCGC. Other aberrant organisms like bacterial ghost inside those cells were also observed (Figure 7B). The abnormally large bacterial developmental forms were similar to those generated after penicillin [41] or IFN-γ treatment [42]. Recently, it has been described the presence of these aberrant bacteria in sphingolipid-depleted cells [14]. A quantitative analysis of chlamydial developmental forms assessed by TEM in cells overexpressing GFP, GFP-Rab14wt, GFP-Rab14S25N and GFP-Rab14ΔGCGC was performed at 24 h p.i. and the results were expressed as a percentage of total bacteria. An increase of morphologically atypical large bacterial forms (LRBs) and bacterial ghosts (GFs) inside inclusions from GFP-Rab14ΔGCGC overexpressing cells was evident (Figure 7C). Moreover, an early bacterial redifferentiation was suggested by a slight increase in EBs in cells overexpressing the negative cytosolic mutant of Rab14. A premature redifferentiation process in sphingomyelin-deficient cells has been recently reported [14].Taken together, the decrease in bacteria number as well as the presence of aberrant bacterial forms inside inclusions suggests an altered chlamydial developmental cycle in cells overexpressing the negative cytosolic mutant of Rab14 and correlates with the reduction of infectious progeny in those cells when analyzed by IFU.

The effect on inclusion size, chlamydial differentiation and yield of infectious particles implies a role for Rab14 in intracellular development and growth of *Chlamydia trachomatis*.

### Sphingolipids are transported to the inclusion via Rab14-positive vesicles

Sphingolipids from the host cell are essential for chlamydial growth and development. However, the molecular machinery underlying bacterial lipid acquisition remains elusive. Several studies have shown that TGN-derived vesicles destined for the plasma membrane are re-directed to the inclusion [9]–[12]. Multivesicular bodies constitute another source for sphingolipids [13]–[15]. Recently, it has been shown that two Rabs, Rab6 and Rab11, are important for an efficient sphingolipid transport to the chlamydial inclusion [21]. Therefore, research focused on whether Rab14, another Golgi-associated Rab, participates in the transport of sphingolipid-containing vesicles from the TGN to the inclusion.

**Figure 8:**
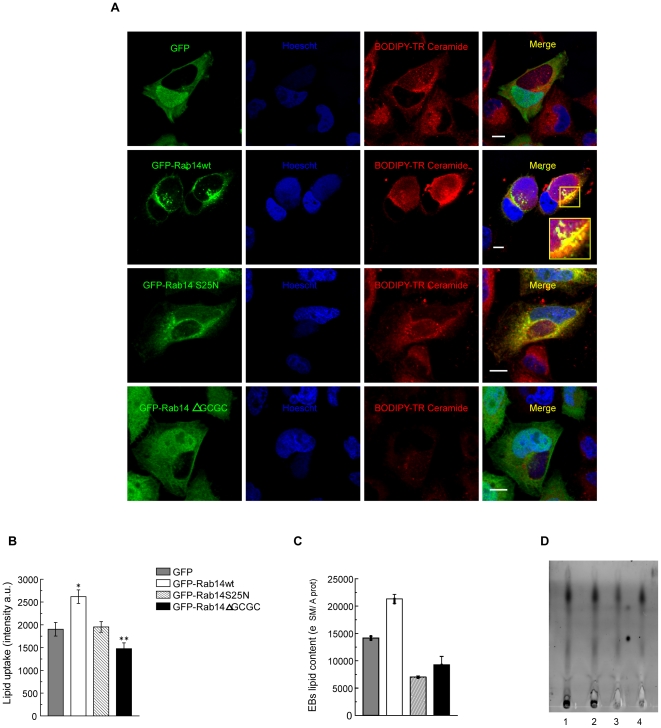
Sphingolipids are transported to chlamydial inclusions via Rab14-positive vesicles. **A**) HeLa cells overexpressing GFP, GFP-Rab14wt, GFP-Rab14S25N or GFP-Rab14ΔGCGC (green) were infected with *C. trachomatis* serovar L2 (MOI 5) for 24 h. Before fixation, cells were incubated 40 minutes with 5 µM BODIPY TR-Ceramide (red) and chased 30 minutes at 37°C. Bacterial DNA was labeled with Hoescht (blue). Inset shows a magnification of the selected area. Bar 10 µm. **B**) Quantification of red fluorescence (lipids) associated with chlamydial inclusions from cells overexpressing GFP, GFP-Rab14wt, GFP-Rab14S25N or GFP-Rab14ΔGCGC infected for 24 h and labeled as indicated in panel A. The red fluorescence intensity of 50 cells in each experimental condition from three independent experiments was measured by confocal microscopy as indicated in Material and Methods. The graph represents media ± sem. Results were statistically analyzed by one-way ANOVA and Tukey post-test (*p<0.01 versus control GFP overexpressing cells; **p<0.001 versus GFP-Rab14wt overexpressing cells). **C**) Fluorescent lipids extracted from EBs purified from transfected cells were measured by spectrofluorometry. Basically, lipid extracts were quantified (530 nm excitation/620 nm emission) and normalized to protein content in the corresponding watery phase (absorbance at 280 nm) measured by spectrophotometry. Emission and absorbance of solvents were subtracted from sample lectures. Data were expressed as a ratio: sphingolipids emission to proteins absorbance. The graph represents media and range of two independent experiments. **D**) Lipid extracts of equal amounts of EBs harvested from GFP, GFP-Rab14wt, GFP-Rab14 S25N or GFP-Rab14 ΔGCGC were resolved by thin layer chromatography as indicated in Material and Methods. Line 1 corresponds to sphingolipid extracts of EBs harvested from GFP, line 2 to GFP-Rab14 wt, line 3 to GFP-Rab14 S25N and line 4 to GFP-Rab14 ΔGCGC. Fluorescent spots were visualized on air-dried plates upon 550 nm excitation. Densitometry was performed using a LAS-4000 EPUV luminometer and LAS image reader software (FUJI Life Science, Japan). A representative TLC plate from two independent experiments is shown.

To examine lipid acquisition, HeLa cells overexpressing GFP alone, GFP-Rab14wt and its negative mutants GFP-Rab14S25N and GFP-Rab14ΔGCGC were infected with *C. trachomatis* for 24 h. Then, cells were labeled for 40 minutes with BODIPY TR Ceramide and chased for 30 minutes prior fixation. BODIPY TR Ceramide is a red fluorescent lipid analogue that turns into labeled sphingolipids in the Golgi apparatus [10]. Confocal images showed an extensive colocalization between GFP-Rab14wt (green) and the sphingolipids (red) at the chlamydial membrane inclusion and at the vesicles found inside the inclusions. In addition, the Golgi apparatus and the majority of the vesicles distributed throughout the cytosol carrying sphingolipids were decorated with GFP-Rab14wt (Figure 8A). However, not all GFP-Rab14 labeled vesicles transport sphingolipids, as well as, not all vesicles carrying sphingolipids are decorated by GFP-Rab14. The lipid uptake was quantified by measuring the red fluorescence intensity (BODIPY TR-labeled sphingolipids) coincident with the chlamydial inclusions as described in Material and Methods. GFP-Rab14wt overeexpressing cells showed a significantly increase in the amount of sphingolipids associated to chlamydial inclusions. In contrast, cells overexpressing the negative cytosolic mutant GFP-Rab14ΔGCGC displayed less red fluorescence intensity associated with the inclusions compared to GFP-Rab14wt overexpressing cells (Figure 8B). Collectively, these data showed that sphingolipid delivery to the maturing chlamydial inclusion was promoted by Rab14. A three-dimensional reconstruction of GFP-Rab14wt overexpressing cells (green) infected with *C. trachomatis* for 24 h (blue) and labeled with fluorescent sphingolipids (red) showed intrainclusion vesicular structures full of lipids decorated by Rab14 in intimate association with the bacteria (Video S2). Sphingolipid uptake was also analyzed in uninfected GFP, GFP-Rab14wt, GFP-Rab14S25N and GFP-Rab14ΔGCGC overexpressing HeLa cells labeled for 40 minutes with BODIPY TR Ceramide and chased for 30 minutes prior fixation. The red fluorescence intensity associated to the entire cells was quantified as indicated in Material and Methods. Overexpression of GFP-Rab14wt increased sphingolipid accumulation whereas the presence of the Rab14 negative mutants diminished sphingolipids inside cells in comparison to control GFP overexpressing cells. Fluorescent sphingolipid accumulation in uninfected cells overexpressing the diverse Rab14 constructs was less evident than in their infected counterparts (Figure S7).To avoid differences due to the ability to accumulate ceramide of host cells, EBs were harvested from GFP, GFP-Rab14wt, GFP-Rab14S25N or GFP-Rab14ΔGCGC overexpressing HeLa cells infected with *C. trachomatis* for 48 h. During the last period of incubation, cells were labeled with BODIPY TR Ceramide for 4 h and chased for an additional 4 h in defatted-BSA enriched cell culture medium to promote fluorescent sphingolipid accumulation in EBs. Cells were examined by LSCM after labeling and back-exchange, and the majority of the fluorescent lipids remained associated with the chlamydial inclusion. After extensive washing to remove unincorporated lipids, cells were lysed and EBs purified. Sphingolipids were extracted from harvested EBs as described in Material and Methods. The fluorescent sphingolipid extracts (SM) were measured by spectrofluorometry (620 nm emission) and normalized to protein content (Prot) in the corresponding watery phase. Protein absorbance was measured at 280 nm in a spectrophotometer. Fluorescence emission and absorbance of the solvents were subtracted from the samples measurements. The ratio SM/Prot was expressed as arbitrary units. The results clearly showed that EBs purified from infected GFP-Rab14wt overexpressing cells were enriched in fluorescent sphingolipids, whereas EBs harvested from infected cells overexpressing both GFP-Rab14 negative mutants had less associated sphingolipids (Figure 8C). These results were further confirmed by thin layer chromatography (TLC). Briefly, EBs were purified from transfected cells labeled with fluorescent sphingolipids. Finally, lipid extracts from harvested EBs were resolved by TLC as described in Material and Methods. A representative TLC plate is shown in Figure 8D. Fluorescent spots were visualized on air-dried plates upon 550 nm excitation. Densitometry was performed using a LAS-4000 EPUV and LAS image reader software (FUJI Life Science, Japan). The amount of sphingolipids from EBs of infected cells overexpressing Rab14 negative mutant was substantially decreased compared to GFP and GFP-Rab14wt overexpressing cells assessed by TLC. The data collected from confocal microscopy images, fluorometric measurements and TLC assays showed that sphingolipid accumulation inside EBs required a functional Rab14.

In summary, these results suggested that *C. trachomatis* actively exploits host Rab14-mediated vesicle transport to deliver TGN-derived lipids to the inclusion. Interruption of this trafficking pathway by overexpression of the negative cytosolic mutant of Rab14 impaired lipid uptake resulting in a delayed inclusion growth, an abnormal chlamydial developmental cycle and an impaired bacteria replication.

These results strengthen that Rab14 is important for sphingolipid transport from the TGN to the inclusion and, thereby, can influence the outcome of *Chlamydia trachomatis* infection.

## Discussion

The success of *Chlamydia trachomatis* development hinges upon the complex host-pathogen interaction. Increasing evidence points out that the invading bacteria subvert intracellular traffic to avoid phagolysosome degradation and to facilitate the delivery of host nutrients to the growing inclusion [3]–[5], [17], [43]. However, the mechanisms involved in these events are poorly defined. It has been demonstrated that *Chlamydia*-containing phagosomes, as soon as they are formed, dissociate from the classical phagocytic/lysosomal pathway and rarely interact with endocytic vesicles [6]. Bacteria actively modify the phagosomal membrane by exclusion or recruitment of selected host proteins. This unique modified phagosome is termed the inclusion. To date, it has been reported that several Rabs are specifically recruited to chlamydial inclusions. Rab4 and Rab11, which are involved in both endosomal receptor recycling and endosome to TGN transport, are found associated with chlamydial inclusions. Additionally, another Golgi-associated GTPases like Rab1, Rab6 and Rab10, are also recruited to chlamydial inclusion membranes [20]–[22]. However, the inclusion is devoid of Rab5 and Rab7, typical Rabs from the endocytic via. The presence of these specific Rabs determines the intracellular fate of the inclusion [5], [17].

Rab14, a GTPase involved in TGN to early endosomes and to plasma membrane transport, is responsible for the maturation block of *Mycobacterium tuberculosis*-containing phagosomes [23]–[26]. Our results demonstrate that *Chlamydia trachomatis* serovar L2 actively recruit host Rab14 in a bacterial protein synthesis-dependent manner but independent of IncA exportation to the chlamydial inclusion membrane. Moreover, microtubule polymerization and Golgi integrity are not required for Rab14 association with the chlamydial inclusions. Coincidently, the recruitment of Rab1, Rab4, Rab6, Rab10 and Rab11 to chlamydial inclusions is maintained in the absence of microtubules [20]. These data are also in agreement with the association of Rab6 and its effector protein BICD1 with the inclusion when the Golgi apparatus is disrupted by BFA treatment [44]. It is important to note that this is the first report showing the recruitment of an endogenous Rab protein to chlamydial inclusions. Furthermore, our results demonstrate that the association to inclusions of endogenous Rab14 does not require an intact Golgi apparatus or microtubule polymerization.

The presence of Rab14 decorating chlamydial inclusions is evident at 10 h p.i. and increases throughout the entire developmental cycle. Mid-stage inclusions (24 h p.i.) show a Rab14-positive rim-like shape surrounding them. At later stages of chlamydial inclusion development, Rab14 label is associated to inclusion membrane but also, is mostly associated to membranous structures within the lumen that resemble intrainclusion vesicles. This surprising finding is in agreement with the observation of translocated lipid droplets inside chlamydial inclusions [15], [16]. The presence of intrainclusion Rab14-labeled vesicular structures reinforces the idea of a general translocation mechanism from the host cell cytoplasm into the inclusion lumen as another strategy for bacterial nutrient acquisition. More detailed microscopy, including immunoelectron microscopy, should be performed to unravel the nature of these vesicle-like structures found inside late stage inclusions of GFP-Rab14wt overexpressing cells. In addition, our results show that Rab14 is required for chlamydial inclusion growth and development, since the presence of its negative cytosolic mutant delays inclusion enlargement.

*Chlamydia trachomatis* undergo a common intracellular biphasic growth cycle, which includes the elementary bodies (EBs), the infectious forms, and the reticulate bodies (RBs) which are the replicative forms. Assessed by electron microscopy, at 24 h p.i., the number of bacteria inside inclusions significantly decreases by overexpression of Rab14 negative mutants. The presence of enlarged RBs and bacterial ghosts is found mainly inside cells overexpressing the negative non-prenylated cytosolic mutant of Rab14. These abnormal chlamydial developmental forms characterized by their ultra-structural morphological alterations are likely aberrant bacteria observed in persistent infections [14], [45]. These atypical chlamydial developmental forms suggest a disturbed bacterial maturation process in cells overexpressing the Rab14 negative cytosolic mutant. Taken together, our results demonstrate that a functional Rab14 is required for a normal chlamydial developmental cycle by promoting bacteria differentiation and replication. At 48 h p.i., the effect of Rab14 negative mutants on *Chlamydia trachomatis* intracellular fate is even more evident. The inclusion forming unit analysis clearly shows that Rab14 promotes bacteria replication and infectivity, since the amount of infectious particles released from GFP-Rab14wt overexpressing cells is greater compared to cells overexpressing both negative mutants of Rab14 or Rab14-depleted cells. The detrimental impact on bacteria infectivity observed after Rab14 silencing or after Rab14 negative mutants overexpression is similar, and consequently, these results confirm a role for Rab14 on bacteria replication. However, we cannot exclude the participation of Rab share effectors that could be sequestered by over-expressed negative mutants of Rab14. Collectively, these data support the idea that bacteria replication and development is favored by the presence of a functional Rab14 at the chlamydial inclusion.

All *Chlamydia* species can accomplish their entire lifecycle, replication and differentiation, within the intracellular inclusion. It has been demonstrated that host cell lipids are essential for bacteria subsistence, growth and multiplication. Lipid droplets translocate to the inclusion lumen and associate with reticular bodies [15], [16]. The late endocytic pathway is implicated in the re-routing of biosynthetic precursors to the chlamydial inclusion via multivesicular bodies [13], [14]. Furthermore, it has been clearly demonstrated that chlamydial inclusion intercepts a subset of TGN-derived vesicles containing sphingolipids and cholesterol and captures these lipids for bacteria benefit [9]–[12]. Recently, it has been shown that Rab6 and Rab11 are required for both chlamydial-induced Golgi fragmentation and sphingolipid transport to the inclusion [21]. This study shows that TGN-derived vesicles full of sphingolipids are enriched in Rab14. In addition, our results show that sphingolipid uptake is increased in uninfected GFP-Rab14wt overexpressing cells whereas sphingolipid accumulation inside cells overexpressing the GFP-Rab14 negative mutants is impaired, confirming a role for Rab14 in sphingolipid transport. Moreover, Rab14 markedly promotes sphingolipid delivery from the TGN to the chlamydial inclusion in infected cells. In contrast, overexpression of the negative cytosolic mutant of Rab14 significantly impairs sphingolipid uptake by chlamydial inclusions quantified by confocal microscopy. EBs harvested from infected cells overexpressing both negative mutants, GFP-Rab14S25N and GFP-Rab14ΔGCGC, accumulate less sphingolipids compared to EBs purified from GFP-Rab14wt or GFP control cells assessed by spectrofluorometry and TLC. Taken together, these data assign a role for Rab14 in sphingolipid transport from the TGN to the inclusion, and consequently, in favouring EBs sphingolipid capture. Recently, it has been shown that host cell sphingolipids are required for inclusion integrity, a normal bacterial developmental cycle and reactivation of persistent infections [14]. Moreover, aberrant bacterial forms are present in sphingomyelin-deficient cells. In agreement, our results show a delayed inclusion enlargement, an impaired bacteria replication and the generation of abnormal bacterial forms in infected cells with reduced sphingolipid acquisition due to the overexpression of the negative mutants of Rab14. Taken together, these results indicate that Rab14, by promoting sphingolipid transport, could contribute to inclusion growth and bacteria development. These and previous data suggest that more than one Rab contributes to lipid transport from the Golgi apparatus to the inclusion and thereby, affects the intracellular development of these bacteria. Thus, *Chlamydia trachomatis* might manipulate functionally redundant intracellular trafficking pathways to ensure their survival and replication. However, the unexpected finding of Rab14-labeled vesicular structures in the lumen of the late stage inclusions has not been reported for another Rab.

In summary, our results suggest a role for Rab14 in inclusion enlargement, sphingolipid transport, chlamydial developmental cycle and bacterial replication. These data could contribute to better delineate the complex molecular machinery used by the bacteria for the acquisition of both energy and nutrients from host cells, and provide insights into bacterial manipulation of vesicular trafficking. This knowledge could help in the development of novel anti-chlamydial therapies. Nevertheless, further experiments should be performed to reveal the chlamydial protein involved in Rab14 recruitment to the inclusion and to elucidate at a deeper level the molecular machinery used by these bacteria to manipulate host cell intracellular trafficking.

## Materials and Methods

### Cells and bacteria

HeLa 229 cells (ABAC, Bs.As., Argentina) were grown in infection medium (IM): D-MEM high glucose (GIBCO-BRL, Bs. As., Argentina) supplemented with 10% fetal bovine serum (FBS) (Internegocios SA, Bs. As., Argentina), 0.3 mg/ml L-glutamine (ICN Biomedicals Inc, Ohaio, USA) and 1.55 mg/ml glucose (Biopack, Bs.As., Argentina). *Chlamydia trachomatis* serovar L2 (gently given and typified by Unidad de Estudios de *Chlamydias*, FFyB, UBA, Bs. As., Argentina) were used. For bacterial propagation, HeLa cells were infected at a multiplicity of infection (MOI) of 20 and incubated at 37°C in an atmosphere of 5% CO2 and 95% humidified air for 48 h. Then, infected cells were lysed with glass beads and EBs were purified by centrifugation as previously described [46]. The purified EBs were suspended in 0.2 M sucrose-5% FBS-0.02 M phosphate buffer (pH = 7.2) and titrated by determination of inclusion forming units (IFU).

### Plasmids and antibodies

The full length human Rab14 cDNA was purchased at the UMR cDNA Resource Center (University of Missouri, USA) and subcloned into the vector pEGFP-C1. The plasmids pEGFP-Rab14S25N and pEGFP-Rab14ΔGCGC were generously provided by Dr. Alfred Nordheim, University of Tuebingen (Tuebingen, Germany). The cDNA of Rab11, a generous gift from Dr. David Sabatini, New York University (New York, USA), was subcloned into the vector pEGFP-C1 as previously reported [47]. The antibodies used in this study were rabbit polyclonal anti-RAB14, mouse monoclonal anti-TGN 46 and mouse monoclonal anti-actin (Abcam, Cambridge, USA), mouse monoclonal anti-β tubulin (Sigma, Bs. As., Argentina), goat anti-mouse HRP-conjugated IgG and goat anti-rabbit HRP-conjugated IgG, donkey anti-rabbit Cy5-labeled IgG, donkey anti-mouse Cy5-conjugated IgG, goat anti-rabbit Texas Red-labeled IgG and goat anti-rabbit FITC-labeled IgG (Jackson Inmunoresearch Laboratories, West Grove, PA, USA and Invitrogen, Bs. As. Argentina). Rabbit polyclonal anti-IncG antibodies were generously provided by Dr. Ted Hackstadt, Laboratory of Intracellular Parasites, National Institute of Allergy and Infectious disease (Rocky Mountain Laboratories, Montana, USA).

### Cell transfection and infection

HeLa 229 cells were grown on 12-mm-diameter glass coverslips in 24-well plates (ETC Internacional, Bs.As., Argentina) until 70% confluence. Cells were washed once with serum-free D-MEM (GIBCO-BRL Bs. As., Argentina) and transfected with Lipofectamine 2000 (Invitrogen, Bs. As., Argentina) using 0.5 µl per 1 µg of DNA per well according to the manufacturer's protocol. Under these conditions, transfected cells ranged between 85% and 95%. Twenty-four hours post-transfection, cells were infected with *C. trachomatis* serovar L2 at a MOI of 5. HeLa cells with bacteria were centrifuged for 10 minutes at 30°C at 1000 rpm and then maintained for two and a half hour at 37°C. After that, cells were washed three times with phosphate-buffered saline (PBS) to eliminate non-internalized bacteria, and finally, cells were incubated in the presence of infection medium (D-MEM without antibiotics) at 37°C in an atmosphere of 5% CO2 and 95% humidified air for the indicated times (post-infection period).

### Reagents and treatments

20 µM Nocodazole (Noc) (Calbiochem, San Diego, CA), 200 µg/ml chloramphenicol (Chlor) (Rontag, Bs. As., Argentina) or 1 µg/ml Brefeldin A (BFA) (Merck, Bs. As., Argentina) were added to infected cells at the indicated post-infection times (p.i.). Then, cells were fixed in 3% p-formaldehyde (PFA) in PBS for 15 min. As mounting medium 0.1 µg/ml Hoescht/Mowiol (Calbiochem and Molecular Probes, USA, respectively) was used.

### Fluorescent labeling and Confocal microscopy

HeLa cells were seeded on coverslips in 24 well plates and infected with *C. trachomatis* according to the assay. For lipid transport experiments, cells were labeled with 5 µM BODIPY TR Ceramide-BSA complex in DMEM (Molecular Probes, USA) for 40 minutes, then the extracellular fluorescent probe was eliminated by extensive washing with cold PBS, and finally, cells were incubated at 37°C for 30 minutes before fixation in 0,03% defatted BSA enriched cell culture medium. Cells from the different experimental conditions were equally and simultaneously processed. Confocal images were captured using the same parameters setting: equal optical magnification (60×) and electronic zoom (2×), identical laser potency (5%), identical photodetector gain (HV 480 V), identical scanning speed (12 µs/pixel). The fixation of the parameters described above determined exposure and acquisition times that were identical for all experimental groups. Then, fluorescence intensity of each channel (green, blue, red and far red) was measured using the FV10-ASW 1.7 Software (Olympus, USA). For lipid uptake quantification, fluorescence intensities of labeled lipid (635 nm laser) were determined by defining regions of interest (ROI) coincident with chlamydial inclusions (405 nm laser) by confocal microscopy and were expressed as arbitrary units (a.u.). Images were acquired at 512 by 512 pixels. The size of inclusions was determined as pixel area assessed by confocal microscopy. The sphingolipid fluorescence intensity was normalized to inclusion area. In uninfected cells, the area of the whole cell was used.

To detect endogenous Rab14 or bacterial IncG, infected cells were fixed in 3% PFA and permeabilized for 20 min with 0.2% saponin/BSA in PBS, then incubated for 1 h with the corresponding primary antibody followed by incubation with a fluorescent-labeled secondary antibody. A similar procedure was used for Golgi apparatus and microtubules staining. Samples were mounted in Hoescht/Mowiol. An Olympus FV-1000 spectral confocal unit mounted on an IX-25 Olympus inverted microscope was used. Confocal images were acquired and analyzed with the FV10-ASW 1.7 Software (Olympus America, Inc., Melville, NY) and then processed using Adobe Photoshop CS3 (Adobe Systems, Inc., San Jose, CA, USA). Adobe Ilustrator CS3, MacBiophotonic Image J and CamStudio softwares were used to perform figures and videos.

### Transmission electron microscopy (TEM)

HeLa cells were grown in T-25 ml flasks and infected with *C. trachomatis* L2 at a MOI of 5 for 24 h. Infected monolayers were fixed with 2% glutaraldehyde/PBS for one hour at 37°C. The cells were removed with 1% gelatin/PBS, gently centrifuged and washed tree times with PBS. After that, cells were incubated with Osmium tetroxide/Potassium ferricyanide/PBS (1∶1∶1) for 90 min. Samples were dehydrated using an increasing acetone series and embedded in Spurr's resin (Ted Pella Inc, USA). Thin sections were cut with an ultramicrotome (Leica ultracut R, Austria) and stained with 1% uranyl acetate and Reynold's lead citrate (Ted Pella Inc, USA) before they were observed with a Zeiss 900 electron microscope (Zeiss, Germany). Images were processed using Adobe Photoshop CS3 (Adobe Systems, Inc., San Jose, CA, USA).

### Inclusion Forming Units (IFU) measurement

Transfected cells, Rab14-depleted and control cells were infected with *C. trachomatis* L2 for 48 h, lysed with glass beads and titrated on fresh HeLa cells. First, cell lysate was centrifuged for 10 min at 500 g to remove debris and progressive dilutions were inoculated onto fresh HeLa cells seeded on a 96 well plate. After 24 h, the number of inclusions formed by chlamydial progeny was assessed by microscopical analysis and expressed as inclusion forming units (IFU) per ml. IFU was normalized to protein determined using the Bradford method.

### Rab14 silencing and western blot

HeLa cells were seeded onto six well tissue culture plates and 24 h later were transfected with 30 ng of All Stars negative control siRNA or 30 ng of a mix of Predesigned siRNA directed against human RAB14 using HiPerFect transfection reagent (Qiagen, Berlin, Germany) and Opti-MEM (Invitrogen, Bs. As., Argentina) according to siRNA transfection protocol at the Qiagen web page (http://www.qiagen.com/transfectionprotocols/default.aspx). At 72 h post transfection, cells were infected with *C. trachomatis*. Two days later, the amount of infectious particles was measured by IFUs. To confirm the decrease of Rab14 protein, cell lysates obtained at 72 h post siRNAs transfection were resolved by SDS-PAGE. Separated proteins were transferred to nitrocellulose membranes and then detected using rabbit polyclonal anti-Rab14 (1∶800) followed by goat anti-rabbit HRP-conjugated antibodies (1∶5000). Protein loading was controlled with mouse monoclonal anti-actin (1∶1000) and goat anti-mouse HRP-labeled antibodies (1∶5000). Amersham ECL Plus™ was used to evince HRP activity (GE Healthcare Life Sciences, Bs.As., Argentina).

### Extraction and quantification of EBs-derived lipids

Transfected cells (T-25 flasks at 80% confluence) were infected with *C. trachomatis* L2 for 48 h. Cells were lysed and EBs were purified as previously described [46]. Prior to EBs harvesting, cells were labeled with 2 µM BODIPY TR Ceramide-BSA complex in DMEM (Molecular Probes, USA) for 4 h, then the extracellular fluorescent probe was eliminated by extensive washing with cold PBS, and finally, cells were incubated at 37°C for 4 h in defatted BSA-enriched medium before EBs harvesting. Lipids from purified EBs were extracted by Bligh and Dyer chloroform:methanol extraction and dried under a stream of Nitrogen [12]. The samples were resuspended in 1∶1 chloroform:methanol and fluorescent lipids were quantified by spectrofluorometry (530 nm excitation/620 nm emission, Packard FluoroCountTM Microplate Fluorometer, USA). Increasing dilutions of BODIPY TR Ceramide were used to generate a curve and fluorescence emission of the solvents was subtracted from sample lectures. Results were normalized to protein content in the corresponding watery phase. Proteins were quantified by their absorbance at 280 nm in a spectrophotometer (Jenway, Genova). Results were expressed as a ratio: lipids emission to proteins absorbance.

### Thin layer chromatography of lipids extracts from EBs

Lipids extracts obtained as indicated above were resolved by thin layer chromatography (TLC). Briefly, lipids from equal amounts of purified EBs were extracted by Bligh and Dyer chloroform:methanol extraction and dried under a stream of Nitrogen [12]. Samples were resuspended in chloroform/methanol/HCl (100∶100∶1, v/v) and resolved on TLC plates using 1-butanol/methanol/acetic acid/water (8∶2∶1∶2, v/v) as solvent system. Fluorescent spots were visualized on air-dried plates upon 550 nm excitation. Densitometry was performed using a LAS-4000 EPUV luminometer and LAS image reader software (FUJI Life Science, Japan).

## Supporting Information

Figure S1 Confocal analysis of a chlamydial inclusion. HeLa cells overexpressing GFP-Rab14wt (green) infected with C. trachomatis serovar L2 (MOI 10) were analyzed by confocal microscopy at 24 p.i. Bacterial DNA was labeled with Hoescht (blue). A) Images show different z-optical planes through the center of the inclusion revealing a fine punctuate pattern of GFP-Rab14 surrounding the chlamydial inclusion. B) Different y sections of the 3-D reconstruction of the z-optical planes showed in panel A.
(0.60 MB TIF)

Figure S2 Intracellular localization of GFP-Rab14 and its mutants. HeLa cells were transfected with pEGFP-Rab14wt, pEGFP-Rab14S25N (a GDP-bound mutant) and pEGFP-Rab14 ΔGCGC (a mutant with its prenylation site deleted). GFP-Rab14wt was found at early endosomes and TGN, GFP-Rab14S25N was retained at the Golgi apparatus whereas GFP-Rab14 ΔGCGC was mostly cytosolic. Bar 10 μm.
(0.22 MB TIF)

Figure S3 Transmission electron microscopy of intrainclusion structures. GFP-Rab14wt overexpressing cells infected with *C. trachomatis* L2 (MOI of 5) were fixed at 48 h p.i. and processed for electron microscopy as indicated in Material and Methods. A magnification of an image shows vesicular membranous structures (arrows) and lipid droplets (asterisk) inside chlamydial inclusions close to bacterial organisms. Bar 1 μm.
(0.79 MB TIF)

Figure S4 GFP-Rab14 distribution in BFA and Nocodazole treated cells. HeLa cells overexpressing GFP, GFP-Rab14 wt, GFP-Rab14 S25N, or GFP-Rab14 ΔGCGC were infected with *C. trachomatis* L2 (MOI 10) and treated with 1 μg/ml Brefeldin A (6 h) (upper panels) or 20 μM Nocodazole (12 h) (lower panels), prior fixation at 24 h p.i. Bacteria were labeled with Hoescht (blue). Mouse monoclonal anti-TGN 46 or mouse monoclonal anti-β-tubulin followed by donkey anti-mouse Cy5-labeled antibodies (1:700) (red) were used to detect Golgi apparatus or microtubules, respectively. The data are representative of at least three independent experiments. Bar 10 μm.
(1.53 MB TIF)

Figure S5 Knockdown of Rab14. HeLa cells were transfected with negative control siRNA or a mix of pre-designed siRNAs against human RAB14 following manufacturer's protocol. At 72 h post transfection, cells were lysed and proteins were separated by SDS-PAGE. Proteins were transferred to nitrocellulose membranes following by immunoblot with rabbit polyclonal anti-Rab14 antibodies (1:800) and goat anti-rabbit HRP-conjugated antibodies (1:5000). Protein loading was controlled with mouse monoclonal anti-actin (1:1000) and goat anti-mouse HRP-labeled antibodies (1:5000). Amersham ECL was used to evince HRP activity.
(0.74 MB TIF)

Figure S6 Efficiency of transfection of HeLa cells. Cells were transfected with pEGFP, pEGFP-Rab14wt, pEGFP-Rab14S25N, or pEGFP-Rab14ΔGCGC as described in Material and Methods. The efficiency of transfection ranged between 85 to 95% with the different plasmids. Confocal images captured at low magnification (60x) show transfected cells in the green channel (GFP-tagged proteins) and the totality of the cells by DIC. Cells observed in both channels were quantified in 10 images from each condition to assess the percentage of GFP-overexpressing cells. DNA was stained with Hoescht (blue).
(5.01 MB TIF)

Figure S7 Lipid uptake in uninfected cells. HeLa cells overexpressing GFP, GFP-Rab14wt, GFP-Rab14S25N or GFP-Rab14ΔGCGC were labeled with 5µM BODIPY TR Ceramide-BSA complex in DMEM (Molecular Probes, USA) for 40 minutes. Then the extracellular fluorescent probe was eliminated by extensive washing with cold PBS, and finally, cells were incubated at 37°C for 30 minutes before fixation in 0,03 % defatted BSA enriched cell culture medium. Sphingolipid accumulation inside cells was quantified as indicated in Material and Methods. Fluorescence intensities of labeled sphingolipids were normalized to the area of whole cells. The graph represents media ± sem. Results were statistically analyzed by one-way ANOVA and Tukey post-test (*p<0.01 and **p<0.001 versus control GFP overexpressing cells).
(0.10 MB TIF)

Video S1 A three-dimensional reconstruction of an infected cell. HeLa cells overexpressing GFP-Rab14wt (green) infected for 48 h with *C. trachomatis* serovar L2 (MOI 5) were reconstructed from consecutive z-sections assessed by confocal microscopy. Inclusion membrane was delimited by labeling with rabbit polyclonal anti-IncG antibodies followed by goat anti-rabbit Texas Red-conjugated IgG (red). Hoescht stained bacterial DNA (blue). Note intrainclusion vesicular structures labeled with GFP-Rab14wt in close contact with the bacteria.
(5.79 MB AVI)

Video S2 Rab14-labeled vesicular structures carry sphingolipids to the inclusion. HeLa cells overexpressing GFP-Rab14wt (green) were infected for 24 h with *C. trachomatis* serovar L2 (MOI 5). Then, cells were labeled for 40 minutes with BODIPY TR Ceramide (red) and chased for 30 minutes prior fixation. Hoescht stained bacterial DNA (blue). Image was reconstructed from consecutive z-sections assessed by confocal microscopy. Note intrainclusion vesicular structures labeled with Rab14wt (green) full of sphingolipids (red) in close contact with bacteria (blue).
(1.85 MB AVI)
