# Supplementary figures and images for: Chlamydia trachomatis Intercepts Golgi-Derived Sphingolipids through a Rab14-Mediated Transport Required for Bacterial Development and Replication
Source: PLoS One. 2010 Nov 22;5(11):e14084. doi: 10.1371/journal.pone.0014084 (PMC2989924; doi:10.1371/journal.pone.0014084)

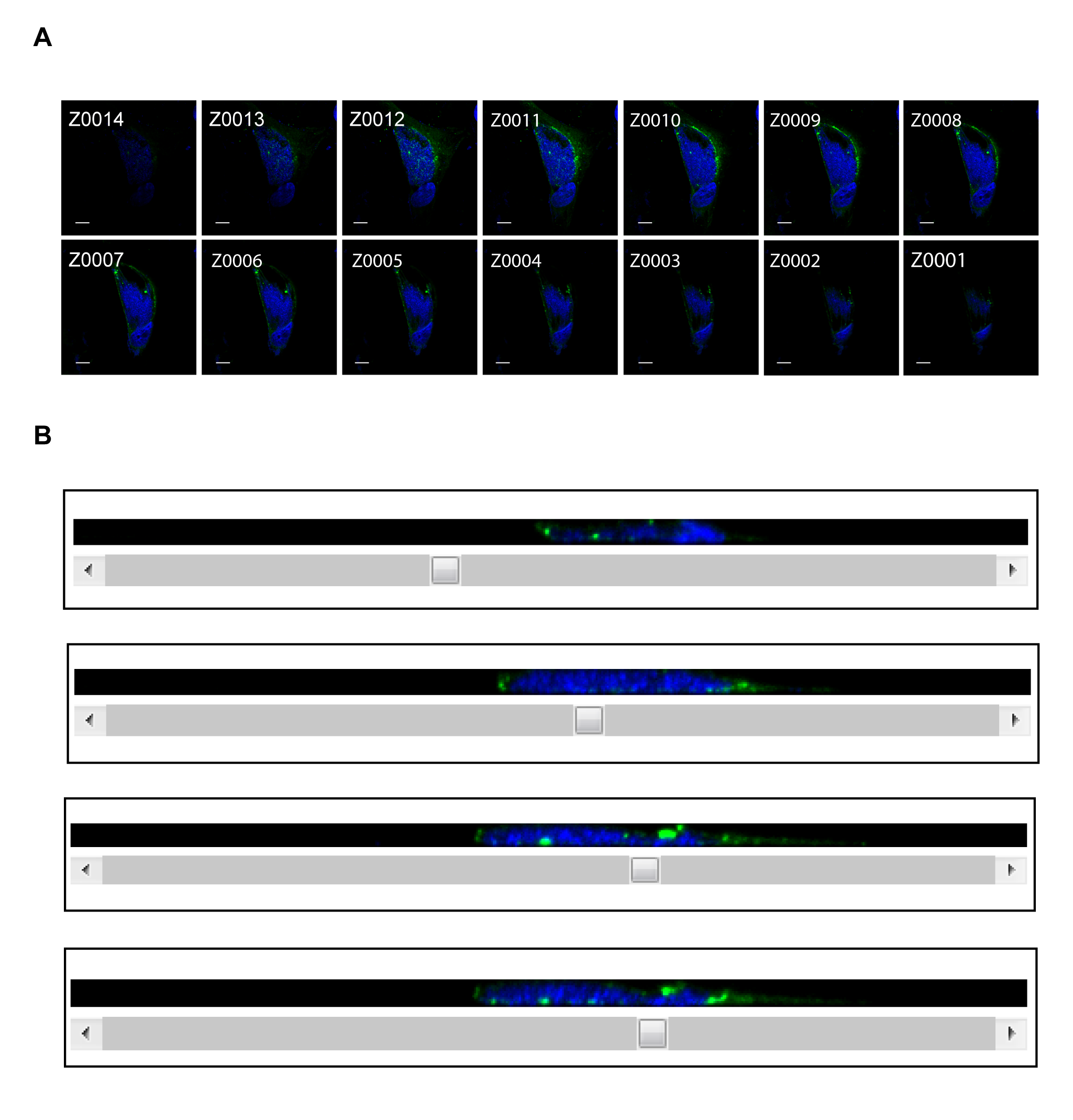

Supplement: Figure S1 — Confocal analysis of a chlamydial inclusion. HeLa cells overexpressing GFP-Rab14wt (green) infected with C. trachomatis serovar L2 (MOI 10) were analyzed by confocal microscopy at 24 p.i. Bacterial DNA was labeled with Hoescht (blue). A) Images show different z-optical planes through the center of the inclusion revealing a fine punctuate pattern of GFP-Rab14 surrounding the chlamydial inclusion. B) Different y sections of the 3-D reconstruction of the z-optical planes showed in panel A. (0.60 MB TIF) [file pone.0014084.s001.tif]

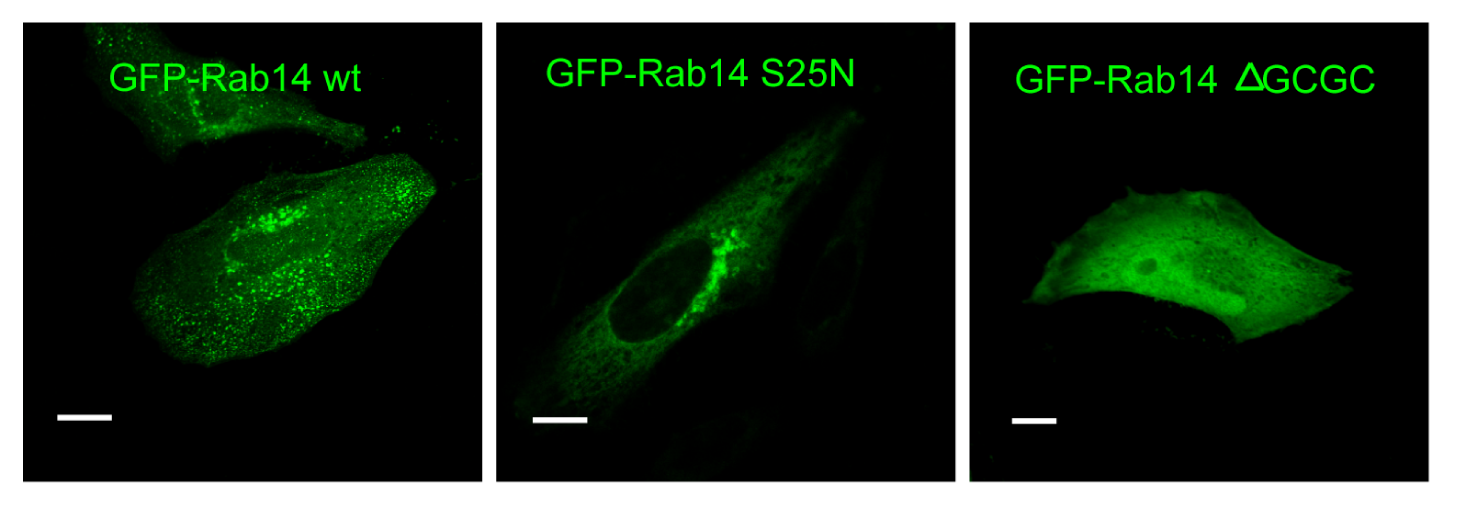

Supplement: Figure S2 — Intracellular localization of GFP-Rab14 and its mutants. HeLa cells were transfected with pEGFP-Rab14wt, pEGFP-Rab14S25N (a GDP-bound mutant) and pEGFP-Rab14 ΔGCGC (a mutant with its prenylation site deleted). GFP-Rab14wt was found at early endosomes and TGN, GFP-Rab14S25N was retained at the Golgi apparatus whereas GFP-Rab14 ΔGCGC was mostly cytosolic. Bar 10 μm. (0.22 MB TIF) [file pone.0014084.s002.tif]

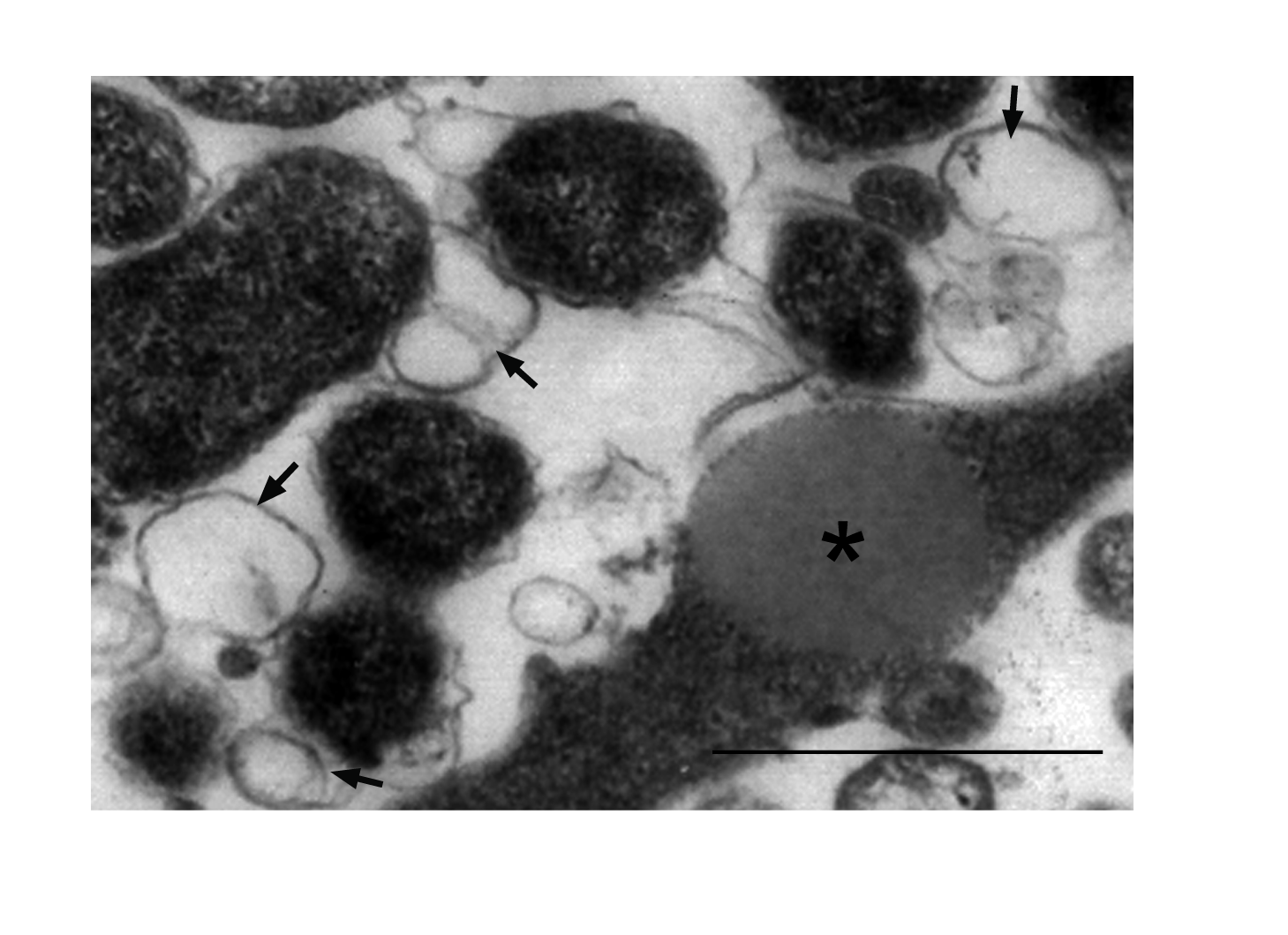

Supplement: Figure S3 — Transmission electron microscopy of intrainclusion structures. GFP-Rab14wt overexpressing cells infected with C. trachomatis L2 (MOI of 5) were fixed at 48 h p.i. and processed for electron microscopy as indicated in Material and Methods. A magnification of an image shows vesicular membranous structures (arrows) and lipid droplets (asterisk) inside chlamydial inclusions close to bacterial organisms. Bar 1 μm. (0.79 MB TIF) [file pone.0014084.s003.tif]

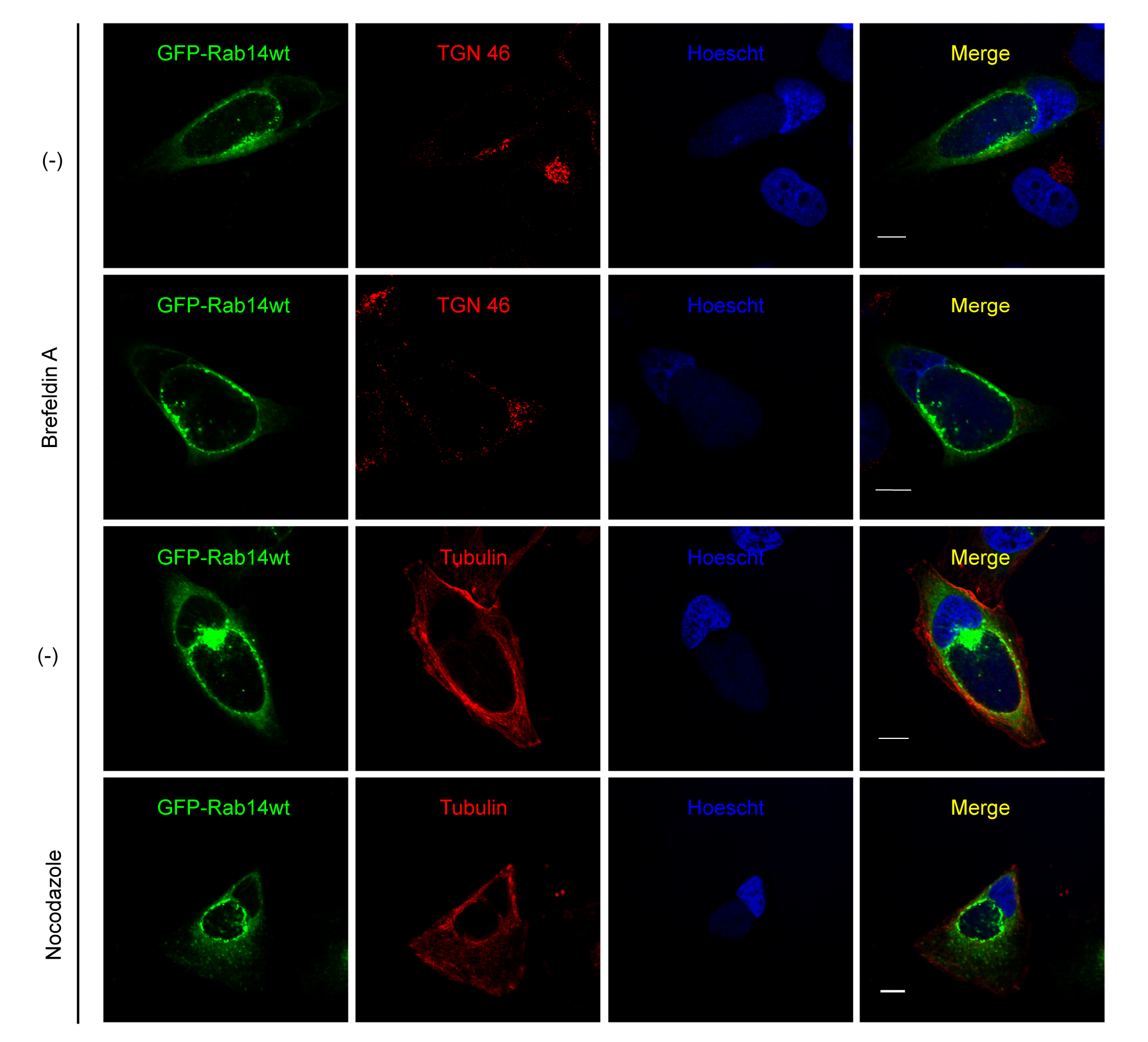

Supplement: Figure S4 — GFP-Rab14 distribution in BFA and Nocodazole treated cells. HeLa cells overexpressing GFP, GFP-Rab14 wt, GFP-Rab14 S25N, or GFP-Rab14 ΔGCGC were infected with C. trachomatis L2 (MOI 10) and treated with 1 μg/ml Brefeldin A (6 h) (upper panels) or 20 μM Nocodazole (12 h) (lower panels), prior fixation at 24 h p.i. Bacteria were labeled with Hoescht (blue). Mouse monoclonal anti-TGN 46 or mouse monoclonal anti-β-tubulin followed by donkey anti-mouse Cy5-labeled antibodies (1:700) (red) were used to detect Golgi apparatus or microtubules, respectively. The data are representative of at least three independent experiments. Bar 10 μm. (1.53 MB TIF) [file pone.0014084.s004.tif]

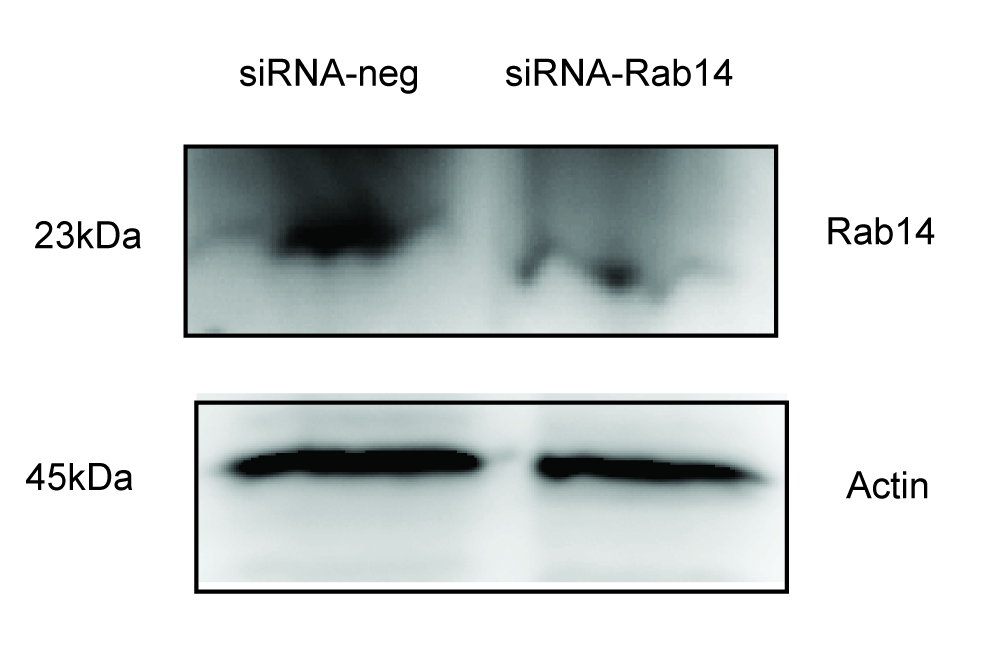

Supplement: Figure S5 — Knockdown of Rab14. HeLa cells were transfected with negative control siRNA or a mix of pre-designed siRNAs against human RAB14 following manufacturer's protocol. At 72 h post transfection, cells were lysed and proteins were separated by SDS-PAGE. Proteins were transferred to nitrocellulose membranes following by immunoblot with rabbit polyclonal anti-Rab14 antibodies (1:800) and goat anti-rabbit HRP-conjugated antibodies (1:5000). Protein loading was controlled with mouse monoclonal anti-actin (1:1000) and goat anti-mouse HRP-labeled antibodies (1:5000). Amersham ECL was used to evince HRP activity. (0.74 MB TIF) [file pone.0014084.s005.tif]

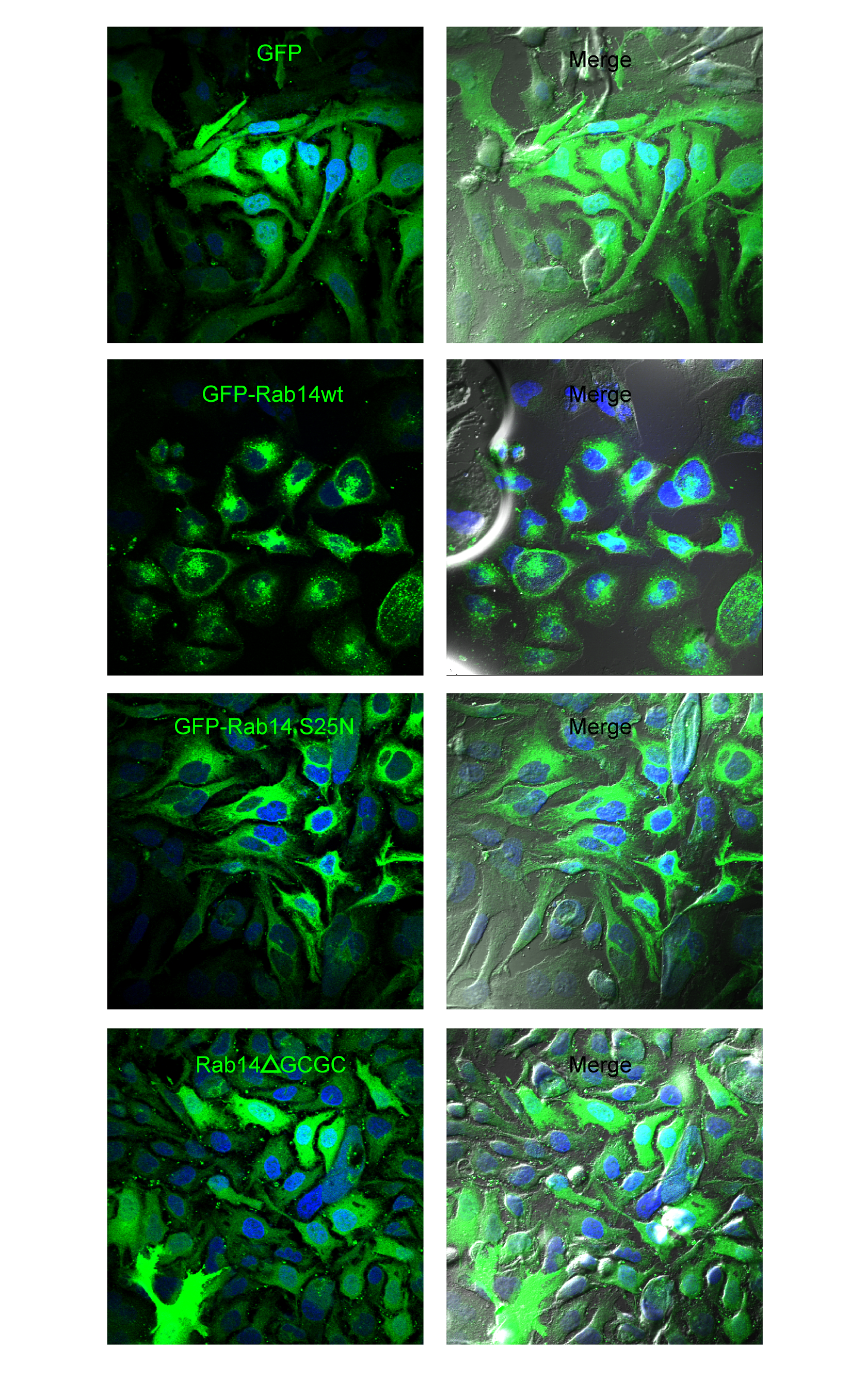

Supplement: Figure S6 — Efficiency of transfection of HeLa cells. Cells were transfected with pEGFP, pEGFP-Rab14wt, pEGFP-Rab14S25N, or pEGFP-Rab14ΔGCGC as described in Material and Methods. The efficiency of transfection ranged between 85 to 95% with the different plasmids. Confocal images captured at low magnification (60x) show transfected cells in the green channel (GFP-tagged proteins) and the totality of the cells by DIC. Cells observed in both channels were quantified in 10 images from each condition to assess the percentage of GFP-overexpressing cells. DNA was stained with Hoescht (blue). (5.01 MB TIF) [file pone.0014084.s006.tif]

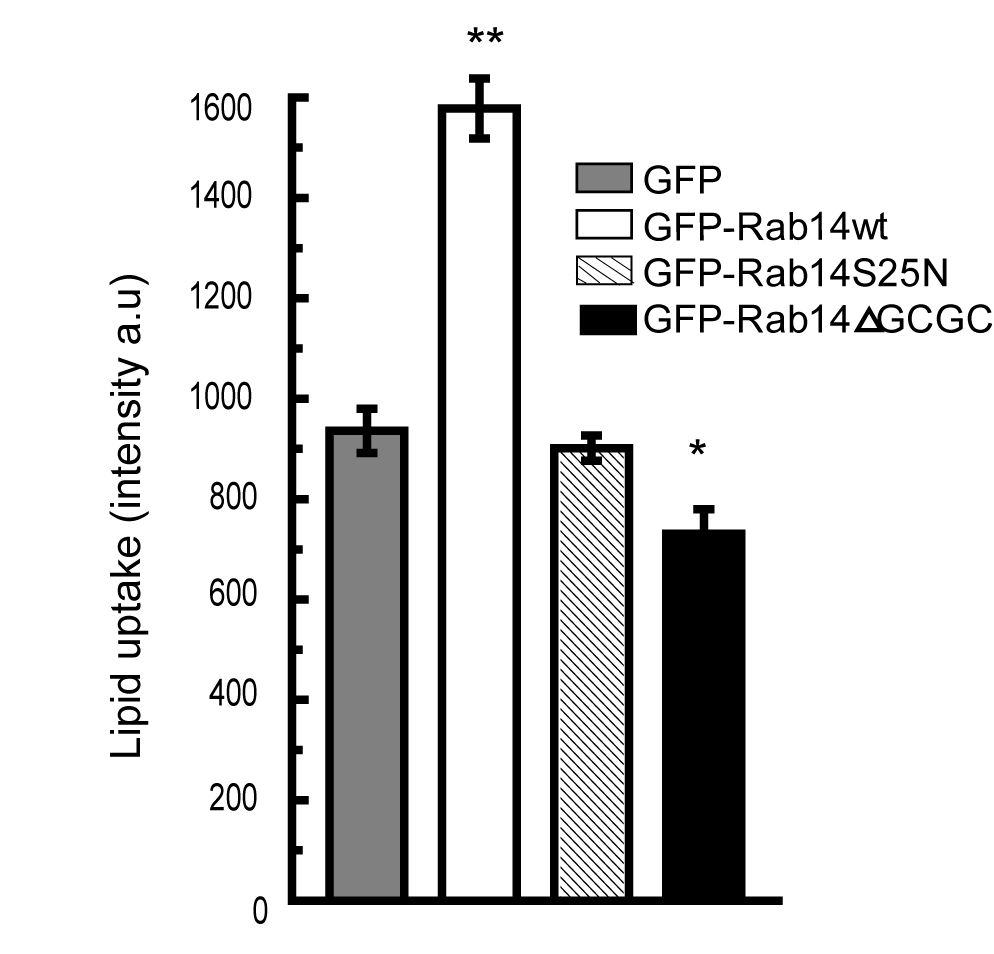

Supplement: Figure S7 — Lipid uptake in uninfected cells. HeLa cells overexpressing GFP, GFP-Rab14wt, GFP-Rab14S25N or GFP-Rab14ΔGCGC were labeled with 5µM BODIPY TR Ceramide-BSA complex in DMEM (Molecular Probes, USA) for 40 minutes. Then the extracellular fluorescent probe was eliminated by extensive washing with cold PBS, and finally, cells were incubated at 37°C for 30 minutes before fixation in 0,03 % defatted BSA enriched cell culture medium. Sphingolipid accumulation inside cells was quantified as indicated in Material and Methods. Fluorescence intensities of labeled sphingolipids were normalized to the area of whole cells. The graph represents media ± sem. Results were statistically analyzed by one-way ANOVA and Tukey post-test (*p<0.01 and **p<0.001 versus control GFP overexpressing cells). (0.10 MB TIF) [file pone.0014084.s007.tif]
